# Exogenous Autoinducer-2 Rescues Intestinal Dysbiosis and Intestinal Inflammation in a Neonatal Mouse Necrotizing Enterocolitis Model

**DOI:** 10.3389/fcimb.2021.694395

**Published:** 2021-08-05

**Authors:** Yan-Chun Ji, Qian Sun, Chun-Yan Fu, Xiang She, Xiao-Chen Liu, Yu He, Qing Ai, Lu-Quan Li, Zheng-Li Wang

**Affiliations:** ^1^Neonatal Diagnosis and Treatment Center of Children’s Hospital of Chongqing Medical University, Chongqing, China; ^2^National Clinical Research Center for Child Health and Disorders, Chongqing, China; ^3^Ministry of Education Key Laboratory of Child Development and Disorders, Chongqing, China; ^4^China International Science and Technology Cooperation Base of Child Development and Critical Disorders, Chongqing, China; ^5^Chongqing Key Laboratory of Pediatrics, Chongqing, China

**Keywords:** necrotizing enterocolitis (NEC), autoinducer-2 (AI-2), intestinal flora, cytokines, intestinal immunity

## Abstract

Autoinducer-2 (AI-2) is believed to be a bacterial interspecies signaling molecule that plays an important role in the regulation of the physiological behaviors of bacteria. The effect of AI-2 on the process of necrotizing enterocolitis (NEC) is unknown, and the aim of this study was to study the effect of AI-2 in a mouse NEC model. C57BL/6 mouse pups were randomly divided into three groups: the control group, the NEC group, and the NEC+AI-2 (NA) group. Exogenous AI-2 (500 nM) was added to the formula milk of the NA group. The concentrations of fecal AI-2 and flora were tested. The expression of cytokines, TLR4 and NF-κB in intestinal tissue was detected. The AI-2 level was significantly decreased in the NEC group (*P*<0.05). Compared with the NEC group, the intestinal injury scores, expression of TLR4, NF-kB, and proinflammatory factors (IL-1β, IL-6, IL-8 and TNF-α) were reduced, and expression of anti-inflammatory factor (IL-10) was increased in the NA group mice (*P*<0.05). At the phylum level, the Proteobacteria abundance in the NA group was significantly increased, while the Bacteroidota abundance in the control group was significantly increased (*P*<0.05). At the genus level, Helicobacter and Clostridium_sensu_stricto_1 exhibited significantly greater abundance in the NEC group than in the other two groups, while Lactobacillus had the opposite trend (*P*<0.05). In addition, the abundances of Klebsiella, Rodentibacter and Enterococcus were significantly higher in the NA group than in the NEC and control groups (*P* < 0.05). Exogenous AI-2 partially reverses flora disorder and decreases inflammation in an NEC mouse model.

## Introduction

Necrotizing enterocolitis (NEC) is a devastating intestinal disease that is a significant cause of death in neonatal intensive care units (Frost et al., 2017; Isani et al., 2018). Five to twelve percent of very low-birth-weight infants were affected by NEC, and surgical intervention was needed in 20-40% of cases (Shulhan et al., 2017). The pathogenesis of NEC is complicated and multifactorial (Niño et al., 2016; Bellodas Sanchez and Kadrofske, 2019), and abnormal microbial colonization and a strong immune response in the gut may be responsible (Neu and Walker, 2011; Raveh-Sadka et al., 2015). The intestinal flora can not only regulate an inflammatory reaction of the intestinal wall through toxic factors but also affect the proliferation, differentiation and gene expression of intestinal wall cells (Hinde and Lewis, 2015). Imbalance in the intestinal flora might disrupt the immune balance of the body, destroy intestinal barrier function, and cause excessive inflammation in intestinal wall tissue, resulting in local or diffuse necrosis (Egan et al., 2016; Shi et al., 2017; Baranowski and Claud, 2019).

Quorum sensing (QS), a microbial cell communication process, dynamically controls different metabolic and physiological activities (Wu et al., 2020). Autoinducer-2 (AI-2), a small molecule, is involved in the QS system, and its production and reaction have been observed throughout the bacterial kingdom (Pereira et al., 2013; Wu et al., 2020). AI-2 is synthesized by many bacteria *via* enzymatic steps. The AI-2 precursor (*S*)-4,5-dihydroxypentane-2,3-dione (DPD) can be synthesized *via* catalytic reactions and spontaneously cyclizes into AI-2 (De Keersmaecker et al., 2005). AI-2 signaling can promote interspecies communication and allow different bacterial species to alter behaviors such as biofilm formation, luminescence and virulence (Cuadra-Saenz et al., 2012; Thompson et al., 2015). AI-2 plays an important role in the colonization of the intestinal flora (Buck et al., 2009). Artificially increasing the level of AI-2 can attenuate antibiotic-induced intestinal dysbiosis, which plays an important role in the development of NEC (Sun et al., 2015). We have previously reported that the AI-2 concentration decreases significantly in the acute phase of NEC and increases gradually in the recovery phase of NEC (Fu et al., 2020). These findings illustrate that AI-2 may be an important target for the correction of intestinal flora imbalance.

Toll-like receptors (TLRs), the classic pattern recognition receptors (PRRs) of the human gut, are able to specifically recognize microbial-associated molecular patterns to induce innate and adaptive immune responses (Kawai and Akira, 2010). An imbalance of the flora may activate TLR4, a major lipopolysaccharide (LPS) receptor in TLRs, and then activate the downstream mediator nuclear factor kappa (NF-κB), further inducing the transcription of various proinflammatory and anti-inflammatory cytokines (Park and Lee, 2013; Mihi and Good, 2019; Van Belkum et al., 2020). TLR4 signaling is an important inflammatory pathway related to bacteria in the development of NEC (Hackam and Sodhi, 2018).

On this background, we hypothesized that AI-2 can regulate the composition of the intestinal flora, correct imbalance of the intestinal flora, and reduce the degree of inflammation in the context of NEC by inhibiting the TLR4/NF-κB signaling pathway. To test this hypothesis, we established an NEC animal model and gavaged the model animals with AI-2 to clarify the influence of AI-2 on the intestinal flora composition and the NEC disease process.

## Materials And Methods

### Induction of Neonatal NEC Mouse Model

The NEC mouse model was constructed according to the methods in previous studies, with some adjustments (Garg et al., 2015; Xiao et al., 2018; Gopalakrishna et al., 2019). Seven-day-old C57BL/6 mouse pups were randomly divided into three groups. The mice in the control group were allowed to nurse their mothers freely; those in the experimental group were fed formula milk (2 g of Similac Advance in 10 ml of 33% Esbilac Puppy Milk Replacer); and those in the intervention group were fed AI-2-containing formula milk (2 g of Similac Advance with 1 ml of 5 μM AI-2 in 9 ml of 33% Esbilac Puppy Milk Replacer), with a final concentration of AI-2 in the formula milk of 500 nM, administered at 30 μl/g body weight *via* a silicone tube (1.9 Fr) every 4 h for 3 days. After the pups in the experimental group and the intervention group were fed the Esbilac formula, they were subjected to asphyxia (100% N2 for 90 s) and cold stress at 4°C (10 min) three times a day for 3 days, while the control pups were not subjected to cold stress or asphyxia. The mice were put back to the cage for relaxation after each feeding or hypoxia-cold stimulation during the modeling period. Three-day NEC modeling was completed after the last feeding. The mice were left to relax for 12 h after feeding, and then intestinal tissues and contents were harvested as test samples. The animal study was reviewed and approved by the Institutional Animal Care and Use Committee at Chongqing Medical University.

### Histology

Three groups of pups were decapitated after NEC induction was completed. The intestine was taken from the body of each mouse, and a 1-cm distal part of the ileum was fixed in paraformaldehyde solution (4%). Then, the sample was dehydrated and embedded in paraffin. Next, the sample was cut into 4-μm slices. Subsequently, the 4-μm tissue slices were stained with hematoxylin and eosin (HE). The degree of NEC was graded by a blinded evaluator using the following standard histological scoring system (Yu et al., 2009): 0, no damage; 1, epithelial cell lifting or separation; 2, moderate villus necrosis; 3, necrosis of the entire villus; and 4, transmural necrosis.

### Immunohistochemistry

Paraffin sections were deparaffinized in xylene and rehydrated with ethanol. The sections were used for immunohistochemistry. The tissue sections were placed in a repair box filled with citric acid (pH 6.0) antigen retrieval buffer and heated for antigen retrieval in a microwave oven for 25 min. Then, the sections were incubated in 3% hydrogen peroxide at room temperature in the dark for 25 min and washed with PBS (pH 7.4). BSA (3%) was added to the sections to evenly cover the tissue, and the tissues were sealed for 30 min. The sealing solution was gently removed, the primary antibody prepared with PBS (pH 7.4) was added to the sections, and the sections were placed flat in a box and incubated overnight at 4°C. The sections were then placed in PBS (pH 7.4) and washed three times. The tissues were covered with an anti-rabbit antibody (HRP-labeled) and incubated at room temperature for 50 min. A chromogenic reaction was performed with DAB color developing solution. The sections were counterstained with hematoxylin stain solution. Staining of the tissues was detected with an optical microscope. Nuclear staining with hematoxylin was blue, and positive staining for DAB was brownish yellow.

### Mouse Intestinal Content Acquisition and AI-2 Activity Measurement

The methods have been previously described (Fu et al., 2020). The ileum and colon of each mouse were irrigated with 400 μl of 2216E liquid medium, and the contents were gathered with sterile tubes, vortexed and centrifuged. The supernatants were filtered through a filter membrane (Millipore, USA), and the filtrates were collected as samples to be tested. The fecal sediment was frozen at -80°C for detection of intestinal flora. A chemically synthesized AI-2 precursor DPD was purchased from Omm Scientific (Dallas, TX, USA). The AI-2 activity in the samples was detected using the *Vibrio harveyi* reporter strain BB170 (Raut et al., 2013). The BB170 strain was grown in 2216E (QDRS BIOTEC, China) liquid medium (30°C for 18 h) and diluted 1:5,000 into fresh 2216E liquid medium. The samples were added to V. harveyi BB170 strain diluent for the AI-2 assay. Additionally, 20 μl of 1 μM AI-2 (Omm Scientific, USA) standard solution, fecal filtrate, and 2216E liquid medium (as a negative control) were added in quintuplicate to a 96-well assay plate (Corning, USA). Then, 180 μl of BB170 diluent was added to a 96-well assay plate to produce a final volume of 200 μl, and the plate was shaken at 30°C and 120 rpm. After 30 min, a BioTek Synergy H1 (USA) instrument was used to measure the bioluminescence intensity every 0.5 h until the value of the negative control group was minimized.

### Fecal Sample Microbiota Analysis

Fecal microbiota genomic DNA was extracted with a QIAamp FAST DNA Stool Mini-Kit (Qiagen, Germany) according to the manufacturer’s instructions, as previously described (Fu et al., 2020). The DNA extracts were detected on 1% agarose gels, and the DNA concentration and purity were assessed with a spectrophotometer (NanoDrop 2000 UV-vis, Thermo Scientific, USA). The V3-V4 hypervariable region of the intestinal bacterial 16S rDNA gene was amplified with the primers 338F (5’-ACTCCTACGGGAGGCAGCAG-3’) and 806R (5’-GGACTACHVGGGTWTCTAAT-3’). The amplification procedure was as follows: initial denaturation (95°C for 3 min); denaturation (95°C for 30 s), annealing (55°C for 30 s) and extension (72°C for 45 s) for a total of 27 cycles; and an additional extension (72°C for 10 min). The product was separated by 2% agarose gel electrophoresis, recovered using an AxyPrep DNA Gel Extraction Kit (Axygen Biosciences) and quantified with a Quantus™ Fluorometer (Promega, USA). A database was created and sequenced on an Illumina MiSeq-related platform. The raw data were processed. Briefly, bases with a quality score <20 were truncated, and sequences with lengths >10 bp overlapped. Reads that exceeded the maximum mismatch ratio of 0.2 in the overlapping region of the splicing sequence were removed. The reads were distinguished according to the primers and barcode, and the sequence direction was adjusted to ensure exact barcode matching. The processed sequences were divided into operational taxonomic units (OTUs) using UPARSE (version 7.1) and OTU clustering of the sequences with a 97% similarity threshold.

### Quantitative Real-Time PCR (qRT-PCR)

RNA was extracted from the ilea of mice and subjected to reverse transcription (RT) with a kit from Takara (Takara, Japan). The resulting cDNA was used for qRT-PCR assays with a TB Green Premix Ex Taq II (Tli RNase H Plus) Kit (Takara, Japan). Glyceraldehyde-3-phosphate dehydrogenase (GAPDH) was used to normalize the input mRNA levels (TLR4, NF-κB, IL-1β, IL-6, IL-8, IL-10, and TNF-α) as an endogenous housekeeping gene. The following primer sequences were used (**Table 1**). The results are shown as the mean 2^−ΔΔCt^ ± SD.

**Table 1:** List of primers.

Gene	Direction	Primers
GAPDH	Forward Reverse	TGAAGCAGGCATCTGAGGG CGAAGGTGGAAGAGTGGGAG
TLR4	Forward Reverse	TTTATTCAGAGCCGTTGGTG CAGAGGATTGTCCTCCCATT
NF-κB	Forward Reverse	ATGTGCATCGGCAAGTGG CAGAAGTTGAGTTTCGGGTAG
IL-1β	Forward Reverse	TGGTGTGTGACGTTCCCATT CAGCACGAGGCTTTTTTGTTG
IL-6	Forward Reverse	CCAAGAGGTGAGTGCTTCCC CTGTTGTTCAGACTCTCTCCCT
IL-8	Forward	CAAGGCTGGTCCATGCTCC
	Reverse	TGCTATCACTTCCTTTCTGTTGC
IL-10	Forward Reverse	GCCGTCATTTTCTGCCTCAT GCTTCCCTATGGCCCTCATT
TNF-α	Forward Reverse	CCAAAGGGATGAGAAGTTCC CTCCACTTGGTGGTTTGCTA

### Enzyme-Linked Immunosorbent Assay (ELISA)

The levels of the inflammatory cytokines IL-1β, IL-6, IL-8, tumor necrosis factor-α (TNF-α) and IL-10 were investigated in the supernatant of mouse intestinal tissue with mouse IL-1β, IL-6, TNF-α and IL-10 ELISA kits (4A BIOTECH, China), respectively, according to the manufacturer’s instructions.

### Western Blotting (WB)

Intestinal tissues were homogenized with an electric homogenizer in NP-40 lysis buffer (Beyotime, China). The concentrations of extracted proteins were measured using a bicinchoninic acid assay kit (Beyotime). The protein solution was mixed with sodium lauryl sulfate sample buffer (Beyotime) at a ratio of 4:1 and then denatured in boiling water for 5 min. The protein samples were separated in a 10% polyacrylamide gel and then transferred onto a polyvinylidene difluoride membrane. The membranes were blocked at room temperature in quick sealing fluid for 10 min and then incubated with antibodies against TLR4, NF-κB and β-actin at 4°C overnight. Subsequently, the membranes were washed and incubated with an anti-rabbit secondary antibody for 2 h. The protein bands were detected using an enhanced chemiluminescence kit (ZENBIO Biotechnology, China) and visualized using a Bio-Rad ChemiDoc™ Touch imaging system. The images were analyzed using the Image Lab and ImageJ software.

### Statistical Analysis

All data were analyzed using GraphPad Prism (Version 8.3.0) and tested for normal distribution. The mean ± standard deviation (SD) was used to describe normally distributed data, and the differences were compared using one-way ANOVA or Student’s t-test. The median and interquartile range (IQR) are used to describe skewed data, and the differences were analyzed by the Kruskal-Wallis test. *P* < 0.05 was considered to indicate statistical significance.

## Results

### AI-2 Reduced the Severity in a Mouse NEC Model

#### Survival Rates

**Figure 1:**
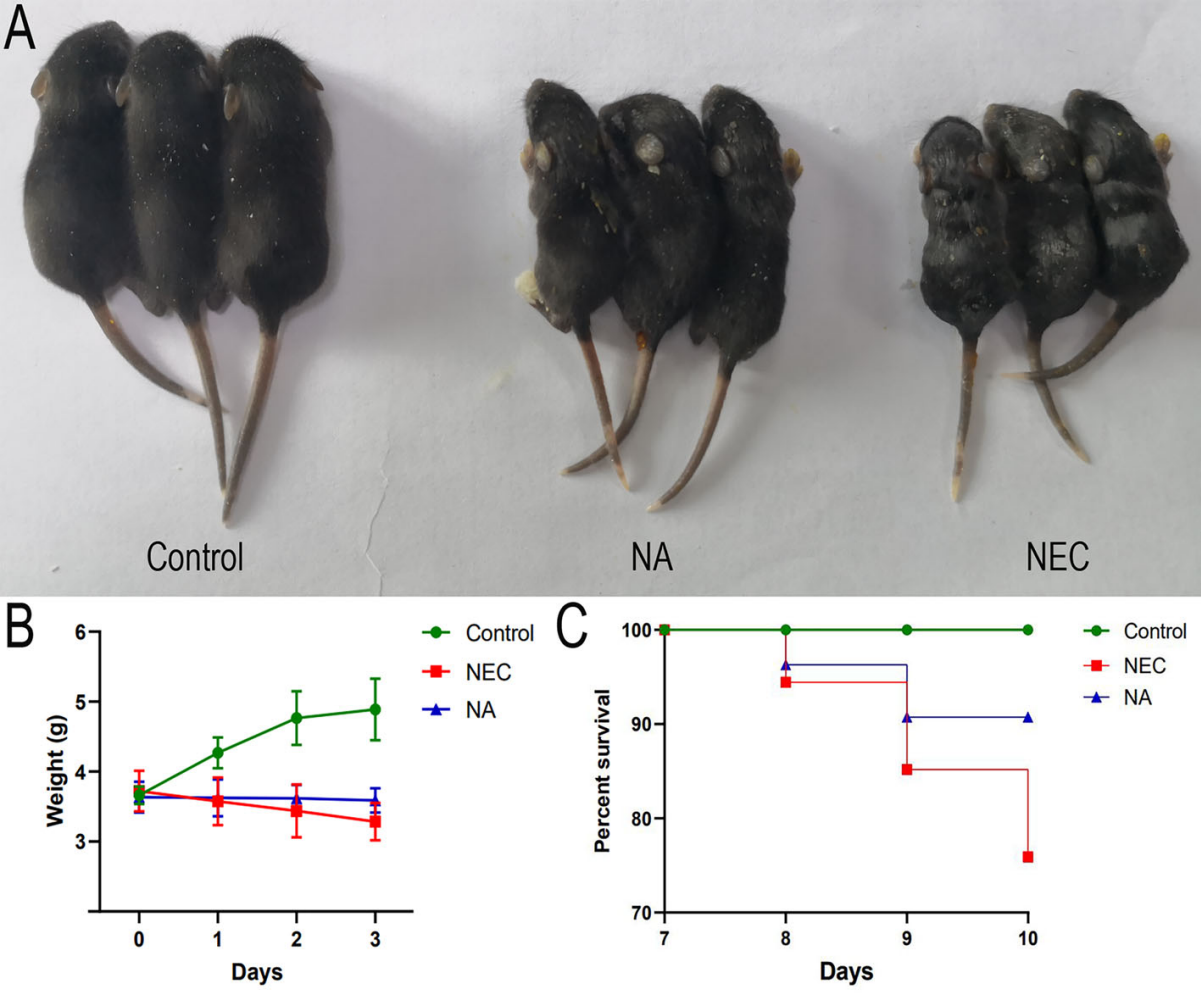
**(A)** The growth of mice in the control group, mice in the NA group and mice in the NEC group. Mice in the NEC group were smaller than those in the control group and NA group. **(B)** Body weight changes of newborn mice in the three groups. Numbers of samples: Con (n=10), NEC (n=10), and NA (n=10). Statistics: two-way ANOVA multiple comparisons method. **(C)** Survival curves of newborn mice in three groups. The survival rate was estimated during modeling, and the results are shown as a Kaplan–Meier plot, with 54 mice in each group at the beginning. Statistics: log-rank (Mantel-Cox) test (*P* < 0.05). Control – normal control, NEC – necrotizing enterocolitis, NA – necrotizing enterocolitis+AI-2.

During modeling, the control group exhibited no deaths, and none of the mice in the NEC group or NA group died before modeling. On the first day of modeling, three deaths occurred in the NEC group, and two deaths occurred in the NA group. On the second day of modeling, five deaths occurred in the NEC group, and three deaths occurred in the NA group. On the third day of modeling, five deaths occurred in the NEC group, and no deaths occurred in the NA group. A significant difference in the final survival rate among the three groups of pups was found: 90.74% (49/54) in the NA group, 75.93% (41/54) in the NEC group, and 100% (54/54) in the control group (*χ2 =* 19.07, *P*=0.0001). The survival curves (**Figure 1C**) of the three groups were significantly different (*P*<0.0001).

#### General Condition and Weight Changes

The neonatal mouse pups in the control group showed good growth and vitality and normal reactivity. The control mice had shiny fur and sufficient subcutaneous fat. The mice in the NEC group began to exhibit abdominal distension, bloating, diarrhea, black stool, and gradually decreased activity. The NA group mice exhibited no black stool and normal activity. The control group mice continued to gain weight during modeling. On days 1, 2, and 3 of NEC modeling, there were no significant differences in weight between the NEC group and the NA group. However, the body weights of the mice in the NEC group decreased during modeling, while the mice in the NA group remained basically unchanged during the modeling period (**Figures 1A, B**). Before sacrifice, the average weight of the mice in the NEC group was significantly lower than that of the mice in the NA group (*P*=0.0157).

#### Macroscopic Appearance of the Gut and Histological Scoring in Model Mice

**Figure 2:**
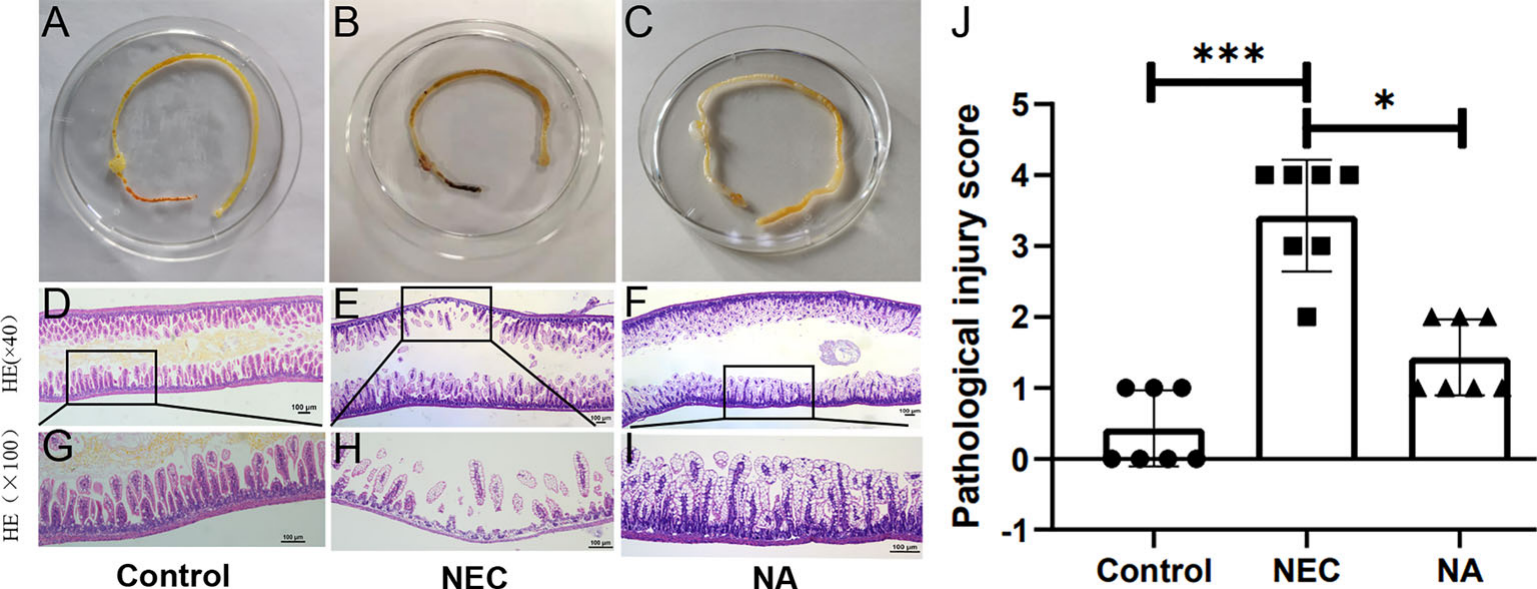
**(A–C)** Macroscopic morphological analyses of the intestines of newborn mice in the three groups. **(D–I)** Images of HE staining by light microscopy. The histological damage in the terminal ilea in the three groups, control group **(D, G)**, NEC group **(E, H)**, NA group **(F, I)**. Black rectangle indicates a representative area, with a zoomed-in image. Magnification: ×40, ×100. scale bar = 100μm. **(J)** Gut histopathological injury scores in the control, NEC and NA group mice. Numbers of samples: Con (n=7), NEC (n=7), and NA (n=7). Statistics: Kruskal-Wallis test (*p < 0.05; ***p < 0.001). Images of HE staining of each sample from the three groups are provided in **Supplementary Figure 1**.

The guts of control mice had no macroscopic damage or pathological changes (**Figure 2A**). NEC mice exhibited obvious injury in the distal ileum, with intestinal gas distension, hemorrhage and discoloration (**Figure 2B**), while NA mice showed obvious gut gas distension and discoloration (**Figure 2C**).

Under an optical microscope, the intestinal tissue structure of the normal control group was clear and complete, with neatly arranged epithelial cells, a thick and continuous muscle layer and no obvious hyperemia, edema or separation in the mucosal layer, submucosa or lamina propria (**Figures 2D, G**). The intestinal tissue of the neonatal mice in the NEC group exhibited a disordered arrangement of epithelial cells, villous degeneration, edema, and partial necrosis, shedding or even disappearance of tissue. The muscle layer was obviously thinned or even broken, and edema was clearly observed in the mucosal layer, submucosa and lamina propria (**Figures 2E, H**). In the NA group, mild to moderate edema, congestion, and villus edema were detected in the gut mucosa and submucosa (**Figures 2F, I**). HE staining of sections from NEC mice showed severe damage and tissue necrosis compared with those of control group and NA group mouse pups, the median intestinal histological score in NA group mice was significantly lower than that in NEC group mice (**Figure 2J**).

### AI-2 Partly Change the Intestinal Flora of NEC

**Figure 3:**
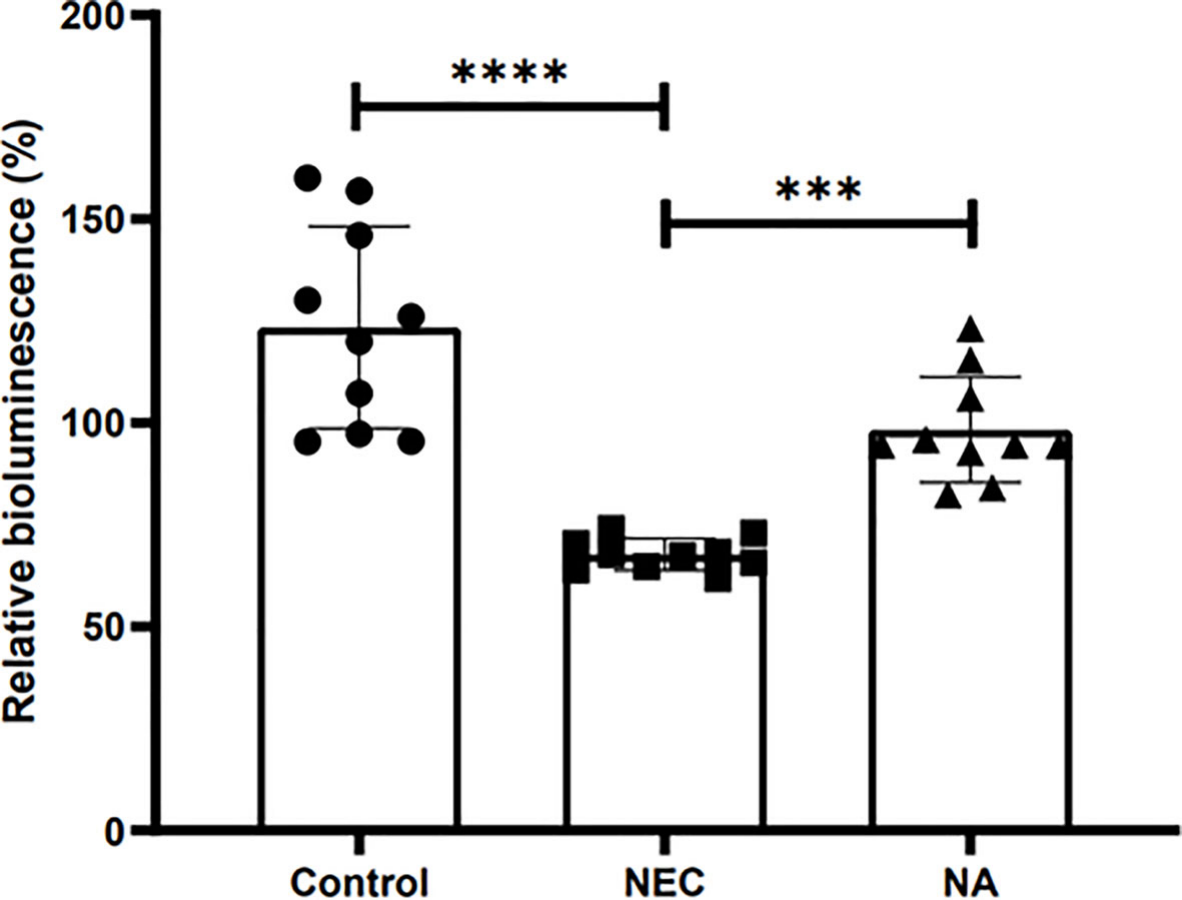
The relative bioluminescence values in the mouse stools of AI-2 in the three groups of newborn mice. Numbers of samples: Con (n=10), NEC (n=10), and NA (n=10). Statistics: one-way ANOVA (***p < 0.001; ****P < 0.0001).

**Figure 4:**
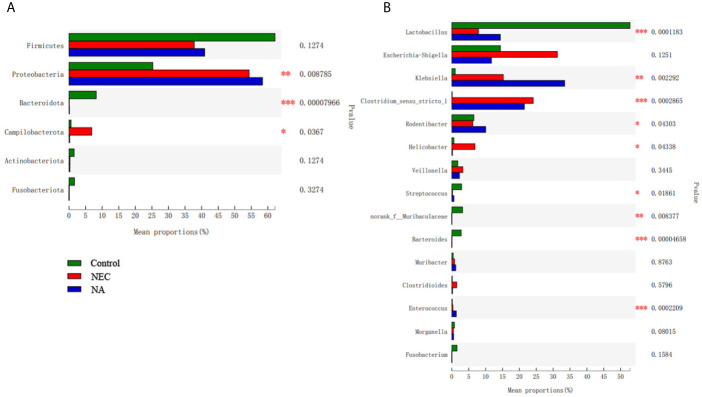
**(A)** The flora composition of mice in the three groups at the phylum level. **(B)** The flora composition of mice in the three groups at the genus level. Numbers of samples: Con (n=12), NEC (n=10), and NA (n=11). Statistics: Kruskal-Wallis test with Scheffe’s *post-hoc* test. * 0.01 < P ≤ 0.05, ** 0.001 < P ≤ 0.01, *** P ≤ 0.001.

**Figure 3** shows the AI-2 levels in the stool of newborn mice in the three groups. The average level in NEC mice was significantly lower than those in NA mice and control mice (*P*<0.05). To investigate the effect of AI-2 on intestinal microbiota in NEC, the proportions of individual taxa in intestinal microbiota were analyzed, and the data are presented in **Figure 4** and **Supplementary Tables 1**, **2**. **Figure 4** shows differences in intestinal microbiota among the three groups. At the phylum level (**Figure 4A**), the average relative abundance of *Firmicutes* was lower in the NEC group than in the NA and control groups (*P*>0.05), while that of *Campilobacterota* was higher in NEC mice than in NA mice and control mice (*P*<0.05). The average relative abundance of *Proteobacteria* was higher in NA mice than in NEC mice and control mice, while that of *Bacteroidota* was greater in control mice than in NEC mice and NA mice (*P*<0.05).

At the genus level (**Figure 4B**), the average abundance of *Lactobacillus* was significantly lower in the NEC group than in the NA group and control group (*P*<0.05). *Clostridium_sensu_stricto_1* and *Helicobacter* were significantly more abundant in the NEC group than in the NA group and control group (*P*<0.05). The abundances of *Klebsiella*, *Enterococcus* and *Rodentibacter* in the NA group were significantly greater than those in the NEC group and control group (*P*<0.05). At the species level (**Supplementary Figure 2**), the average relative abundance of *Clostridium_sensu_stricto_1* was higher in the NEC group than in the other two groups.

Furthermore, differences in the community composition were analyzed by weighted/unweighted UniFrac principal coordinates analysis (PCoA) to discriminate among the control, NEC and NA samples. There were overlaps among the three groups, as shown in **Supplementary Figure 3**.

### AI-2 Reduced the Inflammatory Response

**Figure 5:**
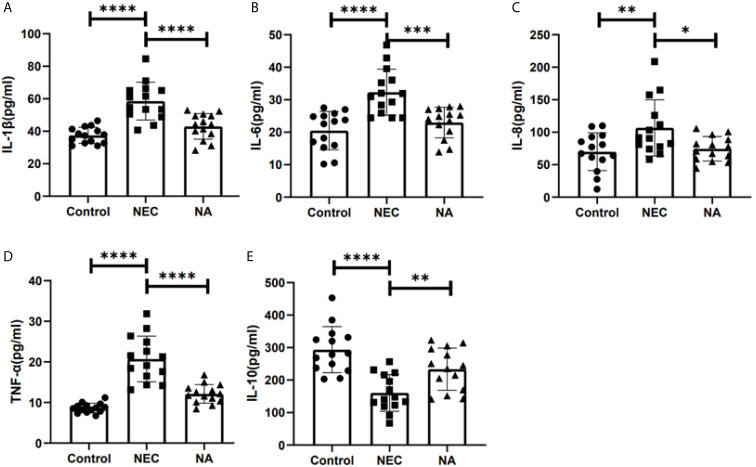
The concentrations of inflammatory cytokines in the three groups were detected by ELISA. **(A)** The concentrations of IL-1β. **(B)** The concentrations of IL-6. **(C)** The concentrations of IL-8. **(D)** The concentrations of TNF-α. **(E)** The concentrations of IL-10. Numbers of samples: Con (n=14), NEC (n=14), and NA (n=14). Statistics: one-way ANOVA (*p < 0.05; **p < 0.01; ***p < 0.001; ****p < 0.0001).

To determine whether cytokine production in NEC mouse intestinal tissue was affected by AI-2, the expression of inflammatory cytokines was tested by qRT-PCR and ELISA. We found that the production of the inflammatory factors IL-1β, IL-6, IL-8, TNF-α and IL-10 in NEC mice was twice, 2.98 times, 1.67 times, 1.88 times and 0.57 times that in NA mice, respectively, and the differences were statistically significant (*P*<0.05). Meanwhile, the expression of proinflammatory factors, including IL-1β (**Figure 5A**), IL-6 (**Figure 5B**), IL-8 (**Figure 5C**) and TNF-α (**Figure 5D**), was significantly lower in NA mice than in NEC mice, and the production of the anti-inflammatory cytokine IL-10 (**Figure 5E**) was significantly increased (*P*<0.05).

**Figure 6:**
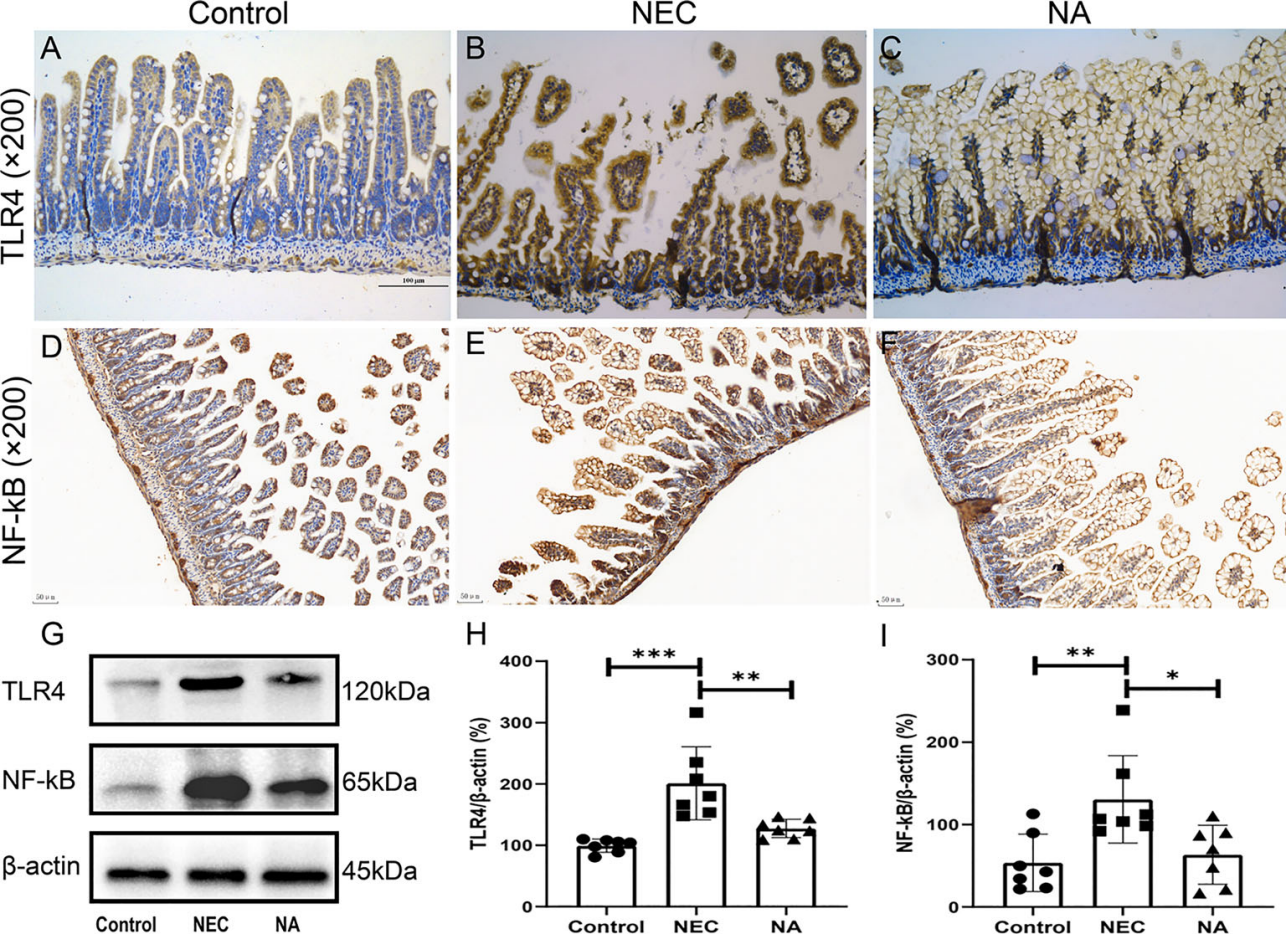
**(A–C)** Expression of the TLR4 and NF-κB **(D–F)** proteins was assessed in intestinal tissues of the three groups by immunohistochemical staining. Magnification: ×200. Scale bars = 100 μm (TLR4) and 50 μm (NF-κB). Images of immunohistochemical staining of the negative control (secondary antibody and DAB control alone) are provided in **Supplementary Figure 4**. **(G–I)** Expression of the TLR4 and NF-κB proteins was determined in intestinal tissues of the three groups by western blotting. Numbers of samples: Con (n=7), NEC (n=7), and NA (n=7). Statistics: Kruskal-Wallis test *P < 0.05, **P < 0.01, ***P < 0.001.

TLR4, which plays a key role in the pathogenesis of NEC, is widely expressed in intestinal epithelial cells and various types of intestinal lymphocytes (Hackam and Sodhi, 2018). After TLR4 is activated by the corresponding pathogenic microorganism, it activates the innate immune response and further activates the downstream NF-κB signaling pathway and mediates the expression and release of the inflammatory factors IL-1, IL-6, IL-8 and TNF-α (Cohen and Prince, 2013). Based on the changes in the levels of inflammatory factors, we further tested the expression of TLR4 and NF-κB in NEC mouse intestinal tissues by qRT-PCR. We found that the transcript expression of TLR4 and NF-kB in NEC mice was 1.73 times and 2.10 times that in NA mice, respectively (*P*<0.05). The TLR4 and NF-κB proteins were qualitatively analyzed by immunohistochemical staining, and we found that staining for both TLR4 (**Figures 6A** **–C**) and NF-κB (**Figures 6D** **–F**) was stronger in the NEC group than in the control and NA groups. Furthermore, we examined the protein expression levels of TLR4 and NF-κB in intestinal tissues by western blotting. **Figure 6G** shows the molecular weight of antibodies. The results showed that TLR4 (**Figure 6H**) and NF-κB (**Figure 6I**) expression in the NEC group was higher than that in the control. However, the expression of these proteins in the NEC group with the supplementation of exogenous AI-2 was lower than that in the NEC group (*P*<0.05).

## Discussion

An important finding of the current study was that exogenous AI-2 supplementation during the NEC modeling process reduced intestinal damage, significantly reduced the expression of TLR4 and related proinflammatory factors, and partially corrected the changes in flora in the NEC mouse model.

### The Relation Between NEC and Intestinal Flora Disorder

Previous studies have shown that the intestinal microbiome is involved in NEC pathogenesis (Neu and Walker, 2011; Raveh-Sadka et al., 2015). Dysbiosis of the intestinal flora existed in the NEC process (Niemarkt et al., 2015). At the phylum level, we found that the relative abundance of *Proteobacteria* was increased, while the relative abundance of *Bacteroides* was decreased in the NEC group. This finding was consistent with a meta-analysis of fecal microorganisms in NEC preterm infants (Pammi et al., 2017). At the genus level, we also found that the abundance levels of *Clostridium_sensu_stricto_1* were significantly increased, and previous studies also found that *Clostridium_sensu_stricto_1* is much more abundant in NEC infants (Fu et al., 2020). The proliferation of Clostridium species in the colon may produce toxins and lead to intestinal epithelial damage (Schönherr-Hellec and Aires, 2019). The abundance levels of *Lactobacillus* in the NEC group were significantly decreased in our study, and other studies indicated that administration of *Lactobacillus* is associated with a significantly decreased risk of NEC (Robertson et al., 2020). Based on these findings, intervention in intestinal flora disorders may be a potential mechanism for the treatment of NEC.

### Exogenous AI-2 Supplementation Partially Reversed the Changes in Intestinal Flora Dysbiosis

AI-2 is an important regulatory factor, which responds to the fluctuations in the microbiota (Wang et al., 2019). Our previous study has shown that changes in AI-2 are associated with the NEC stage. AI-2 levels were reduced in the acute stage and increased in the recovery stage of NEC. The AI-2 concentration inversely correlates with the degree of inflammation and microbial dysbiosis in NEC (Fu et al., 2020). In the present study, a similar phenomenon was observed in the mouse NEC model (**Figure 3**). The AI-2 level was significantly lower in the NEC group than in the other two groups, and intestinal microbiota dysbiosis occurred (**Figures 3**, **4**). The addition of exogenous AI-2 increases the level of AI-2 in the intestine. In the current study, exogenous AI-2 supplementation increased the AI-2 concentration in the NEC mice (**Figure 3**), with a decrease in the inflammatory response. It has been reported that the disruption of the normal microbiota composition by antibiotic treatment leads to a reduction in AI-2 levels and that artificially increasing the concentration of intestinal AI-2 can attenuate the imbalance in intestinal microbiota and the expression of virulence genes (Sun et al., 2015; Thompson et al., 2015; Fu et al., 2020). Interestingly, after exogenous AI-2 supplementation in the present study, the microbiota showed a tendency to partially return to a normal status, which was consistent with the findings reported by Hsiao et al. AI-2 produced by *Ruminococcus obeum* can upregulate the QS system of *Vibrio cholerae*, disrupting its density-sensing regulatory system and leading to the expression of immature virulence factors, thereby reducing the proportion and virulence of *V. cholerae* in the intestine (Hsiao et al., 2014).

Thus, we speculate that multiple factors leading to microbiota dysbiosis significantly decrease the AI-2 concentration, which results in abnormal regulation of the QS system to maintain the stability of the intestinal microbiota, ultimately leading to the occurrence of NEC. Further, the addition of exogenous AI-2 can increase its level in the intestine, which is conducive to the regulation of intestinal homeostasis by the QS system and reduces the inflammatory response and microbiota dysbiosis.

### Exogenous AI-2 Might Reduce Intestinal Inflammation

Studies have shown that the expression of TLR4 in the gut epithelium is increased in human and mouse intestinal inflammation (Leaphart et al., 2007; Egan et al., 2016), and overexpression of TLR4 leads to a signaling cascade that induces nuclear translocation of NF-kB and then promotes overtranscription of proinflammatory cytokines, then leads to the incidence of NEC (Hackam and Sodhi, 2018). Meanwhile, TLR4 inhibitors can relieve inflammation in human and animal NEC models (Niño et al., 2016; Hackam and Sodhi, 2018). In our study, we found that exogenous AI-2 supplementation may promote the expression of the anti-inflammatory factor IL-10 and decrease the expression of proinflammatory factors. Meanwhile, low expression of TLR4 and NF-κB was observed after exogenous AI-2 supplementation. However, no relevant literature to report the cause of this phenomenon in intestine. Therefore, it would be interesting to examine whether exogenous AI-2 could reduce intestinal inflammation in a mouse NEC model by inhibiting TLR4/NF-κB pathway.

In summary, our findings suggest that the administration of exogenous AI-2 may partially reverse the microbiota disorder and decrease inflammation in the mouse NEC model. This study provides new insight into the potential treatment strategy. Further studies are required to ascertain the precise mechanism of the AI-2 effect on NEC.

## Data Availability Statement

Our DNA sequencing data has been uploaded to the NCBI database. The BioProject accession number is PRJNA722686.

## Ethics Statement

The animal study was reviewed and approved by Institutional Animal Care and Use Committee (IACUC) at the University of Chongqing Medical University.

## Author Contributions

L-QL and Z-LW contributed to conceptualization, funding acquisition, and supervision. Y-CJ, QS, C-YF and XS helped with data curation. L-QL, Y-CJ, QS, C-YF, XS, and XC-L worked on the formal analysis. Y-CJ, QS, C-YF, L-QL, XS, and Z-LW carried out the investigation. Y-CJ, QS, C-YF, XS, Z-LW, and QA contributed to the methodology. Y-CJ, QS, C-YF and YH were responsible for project administration and writing the original draft. XS and QA contributed to resources. Z-LW was responsible for the validation. Z-LW and L-QL reviewed and edited the manuscript. All authors contributed to the article and approved the submitted version.

## Funding

This work was supported by the Scientific Research Foundation of the Science and Technology Commission of Chongqing (Grant No. cstc2019jcyj-msxmX0169), the General Basic Research Project from the Ministry of Education Key Laboratory of Child Development and Disorders (Grant No. GBRP-202107), the Chongqing Municipal Administration of Human Resources and Social Security (Grant No. Cx2017107), the National Natural Science Foundation of China (Grant No. 82001602), and the Science and Health Project of the Chongqing Health Commission (Grant No. 2020FYYX217).

## Conflict of Interest

The authors declare that the research was conducted in the absence of any commercial or financial relationships that could be construed as a potential conflict of interest.

## Publisher’s Note

All claims expressed in this article are solely those of the authors and do not necessarily represent those of their affiliated organizations, or those of the publisher, the editors and the reviewers. Any product that may be evaluated in this article, or claim that may be made by its manufacturer, is not guaranteed or endorsed by the publisher.

## References

[B1] Baranowski J. R. Claud E. C. (2019). Necrotizing Enterocolitis and the Preterm Infant Microbiome. Adv. Exp. Med. Biol. 1125, 25–36. 10.1007/5584_2018_313 30680646

[B2] Bellodas Sanchez J. Kadrofske M. (2019). Necrotizing Enterocolitis. Neurogastroenterol Motil. 31 (3), e13569. 10.1111/nmo.13569 30793842

[B3] Buck B. L. Azcarate-Peril M. A. Klaenhammer T. R. (2009). Role of Autoinducer-2 on the Adhesion Ability of Lactobacillus Acidophilus. J. Appl. Microbiol. 107 (1), 269–279. 10.1111/j.1365-2672.2009.04204.x 19302300

[B4] Cohen T. S. Prince A. S. (2013). Activation of Inflammasome Signaling Mediates Pathology of Acute P. Aeruginosa Pneumonia. J. Clin. Invest. 123 (4), 1630–1637. 10.1172/jci66142 23478406 PMC3613922

[B5] Cuadra-Saenz G. Rao D. L. Underwood A. J. Belapure S. A. Campagna S. R. Sun Z.. (2012). Autoinducer-2 Influences Interactions Amongst Pioneer Colonizing Streptococci in Oral Biofilms. Microbiol. (Reading) 158 (Pt 7), 1783–1795. 10.1099/mic.0.057182-0 PMC3542140 22493304

[B6] De Keersmaecker S. C. Varszegi C. van Boxel N. Habel L. W. Metzger K. Daniels R.. (2005). Chemical Synthesis of (S)-4,5-Dihydroxy-2,3-Pentanedione, A Bacterial Signal Molecule Precursor, and Validation of Its Activity in Salmonella Typhimurium. J. Biol. Chem. 280 (20), 19563–19568. 10.1074/jbc.M412660200 15790567

[B7] Egan C. E. Sodhi C. P. Good M. Lin J. Jia H. Yamaguchi Y.. (2016). Toll-Like Receptor 4-Mediated Lymphocyte Influx Induces Neonatal Necrotizing Enterocolitis. J. Clin. Invest. 126 (2), 495–508. 10.1172/jci83356 26690704 PMC4731173

[B8] Frost B. L. Modi B. P. Jaksic T. Caplan M. S. (2017). New Medical and Surgical Insights Into Neonatal Necrotizing Enterocolitis: A Review. JAMA Pediatr. 171 (1), 83–88. 10.1001/jamapediatrics.2016.2708 27893069

[B9] Fu C. Y. Li L. Q. Yang T. She X. Ai Q. Wang Z. L. (2020). Autoinducer-2 May Be a New Biomarker for Monitoring Neonatal Necrotizing Enterocolitis. Front. Cell Infect. Microbiol. 10, 140. 10.3389/fcimb.2020.00140 32373545 PMC7179697

[B10] Garg P. M. Tatum R. Ravisankar S. Shekhawat P. S. Chen Y. H. (2015). Necrotizing Enterocolitis in a Mouse Model Leads to Widespread Renal Inflammation, Acute Kidney Injury, and Disruption of Renal Tight Junction Proteins. Pediatr. Res. 78 (5), 527–532. 10.1038/pr.2015.146 26270572 PMC4628581

[B11] Gopalakrishna K. P. Macadangdang B. R. Rogers M. B. Tometich J. T. Firek B. A. Baker R.. (2019). Maternal IgA Protects Against the Development of Necrotizing Enterocolitis in Preterm Infants. Nat. Med. 25 (7), 1110–1115. 10.1038/s41591-019-0480-9 31209335 PMC7424541

[B12] Hackam D. J. Sodhi C. P. (2018). Toll-Like Receptor-Mediated Intestinal Inflammatory Imbalance in the Pathogenesis of Necrotizing Enterocolitis. Cell Mol. Gastroenterol. Hepatol 6 (2), 229–238.e221. 10.1016/j.jcmgh.2018.04.001 30105286 PMC6085538

[B13] Hinde K. Lewis Z. T. (2015). MICROBIOTA. Mother’s Littlest Helpers. Science 348 (6242), 1427–1428. 10.1126/science.aac7436 26113704

[B14] Hsiao A. Ahmed A. M. Subramanian S. Griffin N. W. Drewry L. L. Petri W. A. Jr.. (2014). Members of the Human Gut Microbiota Involved in Recovery From Vibrio Cholerae Infection. Nature 515 (7527), 423–426. 10.1038/nature13738 25231861 PMC4353411

[B15] Isani M. A. Delaplain P. T. Grishin A. Ford H. R. (2018). Evolving Understanding of Neonatal Necrotizing Enterocolitis. Curr. Opin. Pediatr. 30 (3), 417–423. 10.1097/mop.0000000000000629 29601338

[B16] Kawai T. Akira S. (2010). The Role of Pattern-Recognition Receptors in Innate Immunity: Update on Toll-Like Receptors. Nat. Immunol. 11 (5), 373–384. 10.1038/ni.1863 20404851

[B17] Leaphart C. L. Cavallo J. Gribar S. C. Cetin S. Li J. Branca M. F.. (2007). A Critical Role for TLR4 in the Pathogenesis of Necrotizing Enterocolitis by Modulating Intestinal Injury and Repair. J. Immunol. 179 (7), 4808–4820. 10.4049/jimmunol.179.7.4808 17878380

[B18] Mihi B. Good M. (2019). Impact of Toll-Like Receptor 4 Signaling in Necrotizing Enterocolitis: The State of the Science. Clin. Perinatol 46 (1), 145–157. 10.1016/j.clp.2018.09.007 30771815 PMC6383801

[B19] Neu J. Walker W. A. (2011). Necrotizing Enterocolitis. N. Engl. J. Med. 364 (3), 255–264. 10.1056/NEJMra1005408 21247316 PMC3628622

[B20] Niemarkt H. J. de Meij T. G. van de Velde M. E. van der Schee M. P. van Goudoever J. B. Kramer B. W.. (2015). Necrotizing Enterocolitis: A Clinical Review on Diagnostic Biomarkers and the Role of the Intestinal Microbiota. Inflamm. Bowel Dis. 21 (2), 436–444. 10.1097/mib.0000000000000184 25268636

[B21] Niño D. F. Sodhi C. P. Hackam D. J. (2016). Necrotizing Enterocolitis: New Insights Into Pathogenesis and Mechanisms. Nat. Rev. Gastroenterol. Hepatol 13 (10), 590–600. 10.1038/nrgastro.2016.119 27534694 PMC5124124

[B22] Pammi M. Cope J. Tarr P. I. Warner B. B. Morrow A. L. Mai V.. (2017). Intestinal Dysbiosis in Preterm Infants Preceding Necrotizing Enterocolitis: A Systematic Review and Meta-Analysis. Microbiome 5 (1), 31. 10.1186/s40168-017-0248-8 28274256 PMC5343300

[B23] Park B. S. Lee J. O. (2013). Recognition of Lipopolysaccharide Pattern by TLR4 Complexes. Exp. Mol. Med. 45 (12), e66. 10.1038/emm.2013.97 24310172 PMC3880462

[B24] Pereira C. S. Thompson J. A. Xavier K. B. (2013). AI-2-Mediated Signalling in Bacteria. FEMS Microbiol. Rev. 37 (2), 156–181. 10.1111/j.1574-6976.2012.00345.x 22712853

[B25] Raut N. Pasini P. Daunert S. (2013). Deciphering Bacterial Universal Language by Detecting the Quorum Sensing Signal, Autoinducer-2, With a Whole-Cell Sensing System. Anal. Chem. 85 (20), 9604–9609. 10.1021/ac401776k 24047052

[B26] Raveh-Sadka T. Thomas B. C. Singh A. Firek B. Brooks B. Castelle C. J.. (2015). Gut Bacteria are Rarely Shared by Co-Hospitalized Premature Infants, Regardless of Necrotizing Enterocolitis Development. Elife 4, e05477. 10.7554/eLife.05477 PMC4384745 25735037

[B27] Robertson C. Savva G. M. Clapuci R. Jones J. Maimouni H. Brown E.. (2020). Incidence of Necrotising Enterocolitis Before and After Introducing Routine Prophylactic Lactobacillus and Bifidobacterium Probiotics. Arch. Dis. Child Fetal Neonatal Ed 105 (4), 380–386. 10.1136/archdischild-2019-317346 31666311 PMC7363787

[B28] Schönherr-Hellec S. Aires J. (2019). Clostridia and Necrotizing Enterocolitis in Preterm Neonates. Anaerobe 58, 6–12. 10.1016/j.anaerobe.2019.04.005 30980889

[B29] Shi N. Li N. Duan X. Niu H. (2017). Interaction Between the Gut Microbiome and Mucosal Immune System. Mil Med. Res. 4, 14. 10.1186/s40779-017-0122-9 28465831 PMC5408367

[B30] Shulhan J. Dicken B. Hartling L. Larsen B. M. (2017). Current Knowledge of Necrotizing Enterocolitis in Preterm Infants and the Impact of Different Types of Enteral Nutrition Products. Adv. Nutr. 8 (1), 80–91. 10.3945/an.116.013193 28096129 PMC5227976

[B31] Sun Z. Grimm V. Riedel C. U. (2015). AI-2 to the Rescue Against Antibiotic-Induced Intestinal Dysbiosis? Trends Microbiol. 23 (6), 327–328. 10.1016/j.tim.2015.04.002 25900476

[B32] Thompson J. A. Oliveira R. A. Djukovic A. Ubeda C. Xavier K. B. (2015). Manipulation of the Quorum Sensing Signal AI-2 Affects the Antibiotic-Treated Gut Microbiota. Cell Rep. 10 (11), 1861–1871. 10.1016/j.celrep.2015.02.049 25801025

[B33] Van Belkum M. Mendoza Alvarez L. Neu J. (2020). Preterm Neonatal Immunology at the Intestinal Interface. Cell Mol. Life Sci. 77 (7), 1209–1227. 10.1007/s00018-019-03316-w 31576423 PMC11105006

[B34] Wang Y. Liu B. Grenier D. Yi L. (2019). Regulatory Mechanisms of the LuxS/AI-2 System and Bacterial Resistance. Antimicrob. Agents Chemother. 63(10), e01186–19. 10.1128/aac.01186-19 PMC6761564 31383657

[B35] Wu S. Liu J. Liu C. Yang A. Qiao J. (2020). Quorum Sensing for Population-Level Control of Bacteria and Potential Therapeutic Applications. Cell Mol. Life Sci. 77 (7), 1319–1343. 10.1007/s00018-019-03326-8 31612240 PMC11104945

[B36] Xiao S. Li Q. Hu K. He Y. Ai Q. Hu L.. (2018). Vitamin A and Retinoic Acid Exhibit Protective Effects on Necrotizing Enterocolitis by Regulating Intestinal Flora and Enhancing the Intestinal Epithelial Barrier. Arch. Med. Res. 49 (1), 1–9. 10.1016/j.arcmed.2018.04.003 29699808

[B37] Yu X. Radulescu A. Zorko N. Besner G. E. (2009). Heparin-Binding EGF-Like Growth Factor Increases Intestinal Microvascular Blood Flow in Necrotizing Enterocolitis. Gastroenterology 137 (1), 221–230. 10.1053/j.gastro.2009.03.060 19361505 PMC2704259

