# Supplementary material for: Exogenous Autoinducer-2 Rescues Intestinal Dysbiosis and Intestinal Inflammation in a Neonatal Mouse Necrotizing Enterocolitis Model
Source: Front Cell Infect Microbiol. 2021 Aug 5;11:694395. doi: 10.3389/fcimb.2021.694395 (PMC8375469; doi:10.3389/fcimb.2021.694395)
Supplement: Supplementary file 1 [file Image_1.pdf]

Supplementary Material

1.1 Supplementary Figures

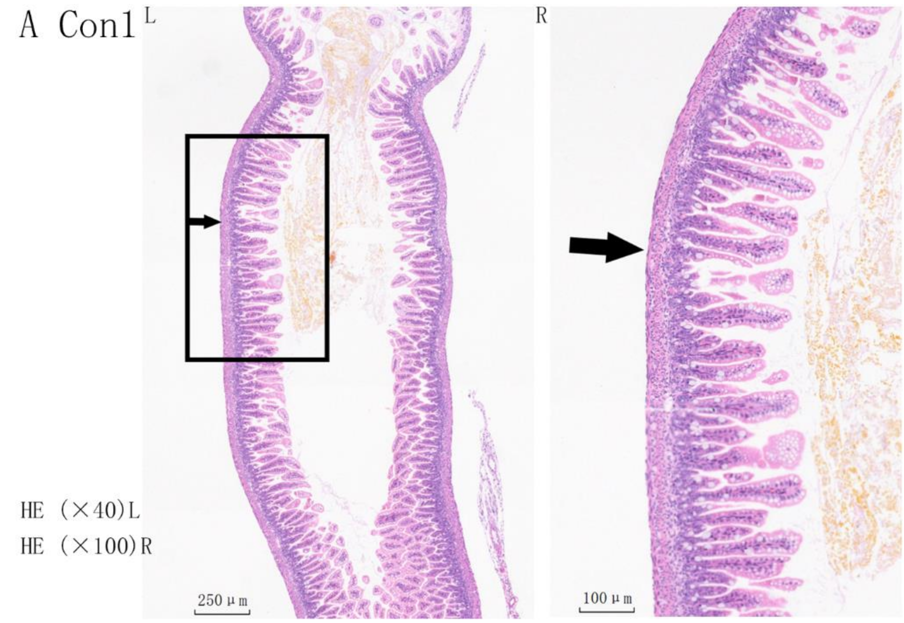

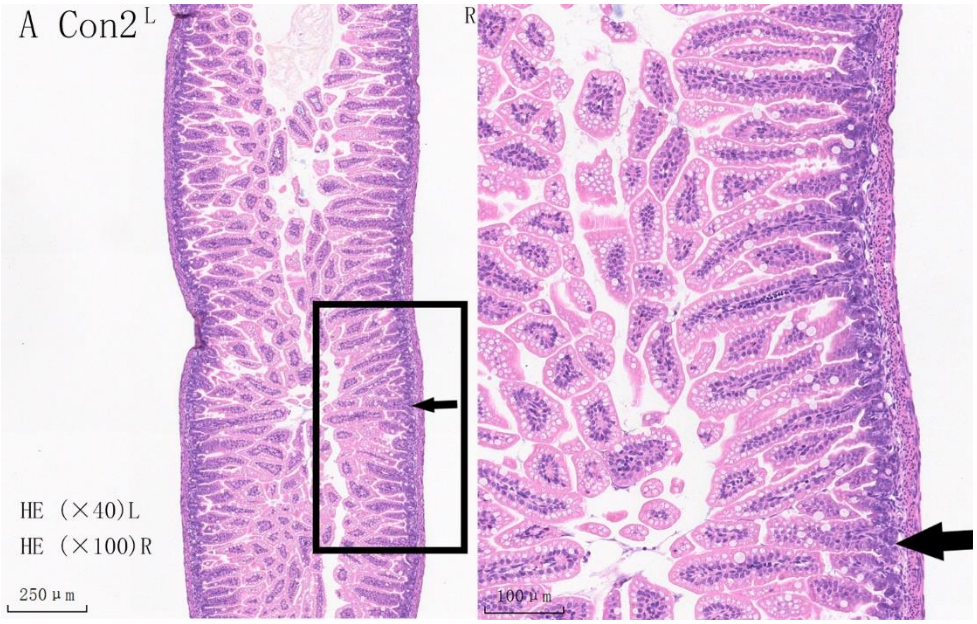

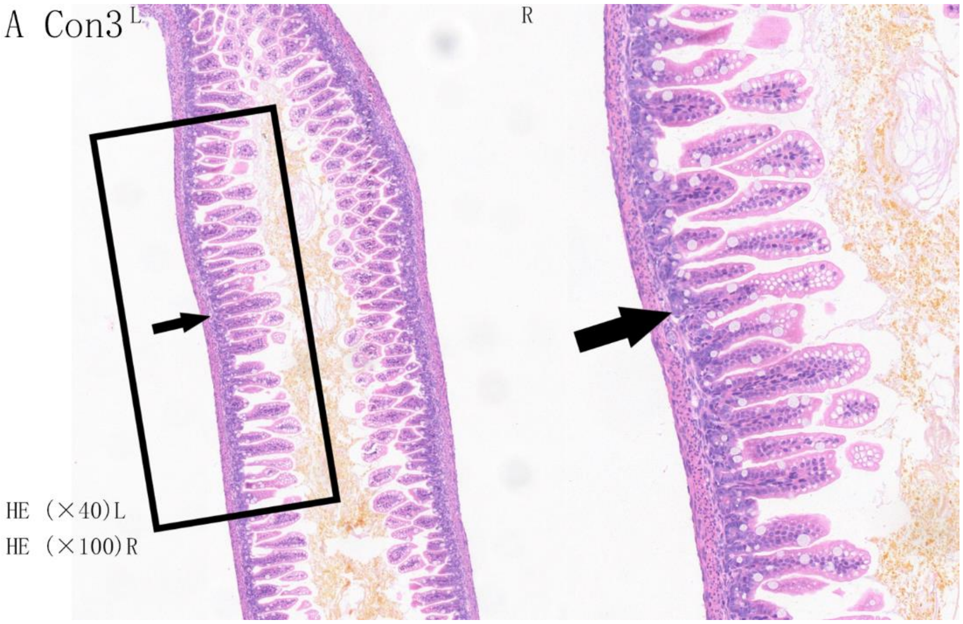
A Con3
L
R
HE (×40)L
HE (×100)R

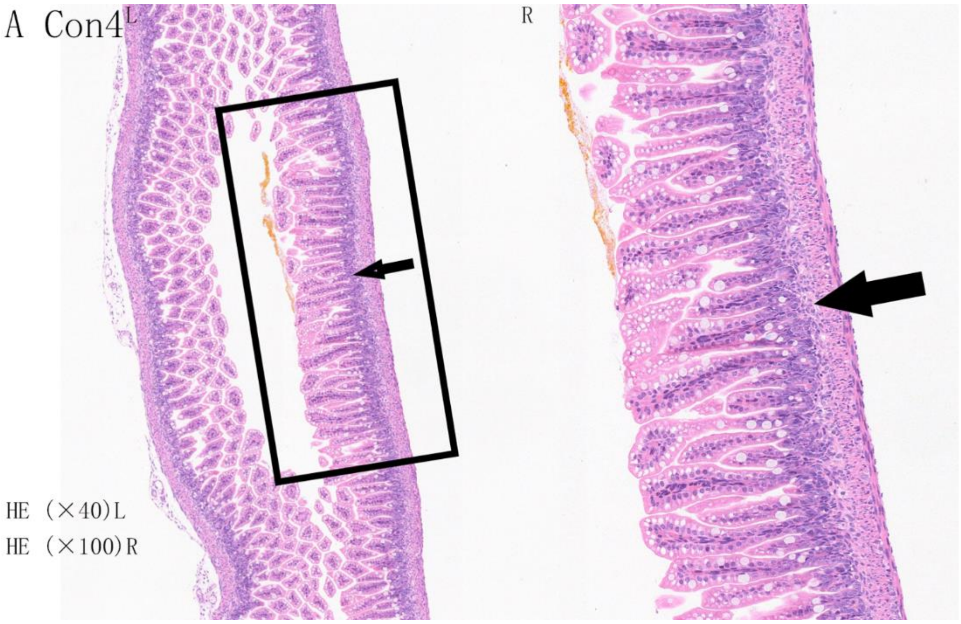
A Con4
L
R
HE (×40)L
HE (×100)R

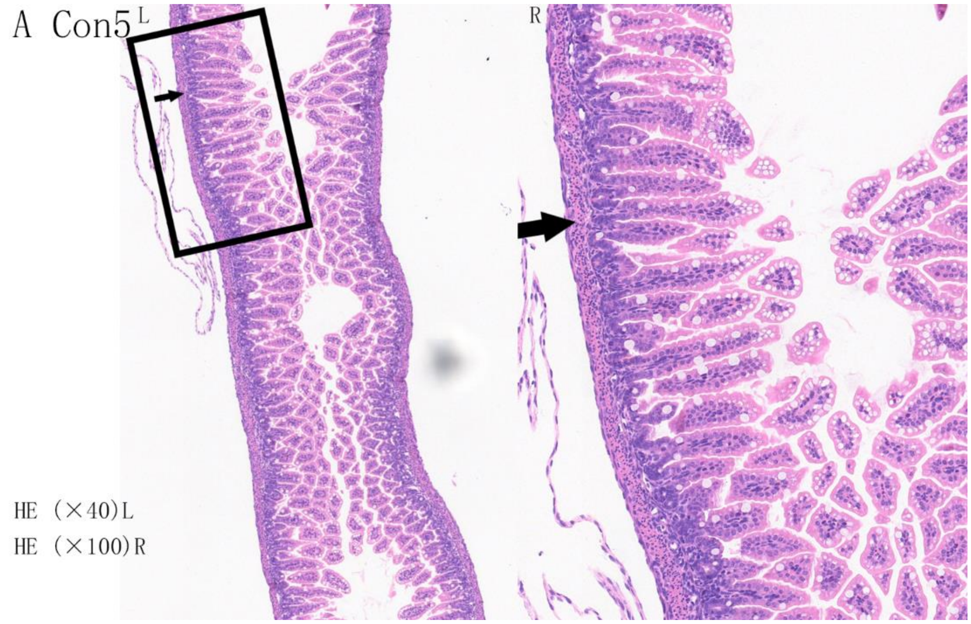
A Con5 L
R
HE (×40)L
HE (×100)R

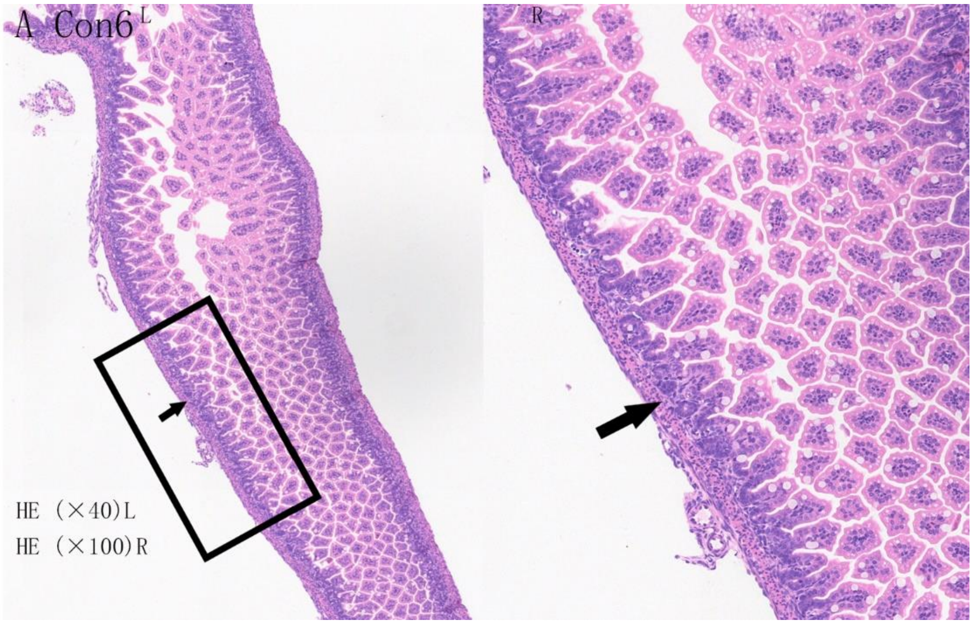
A Con6 L
R
HE (×40)L
HE (×100)R

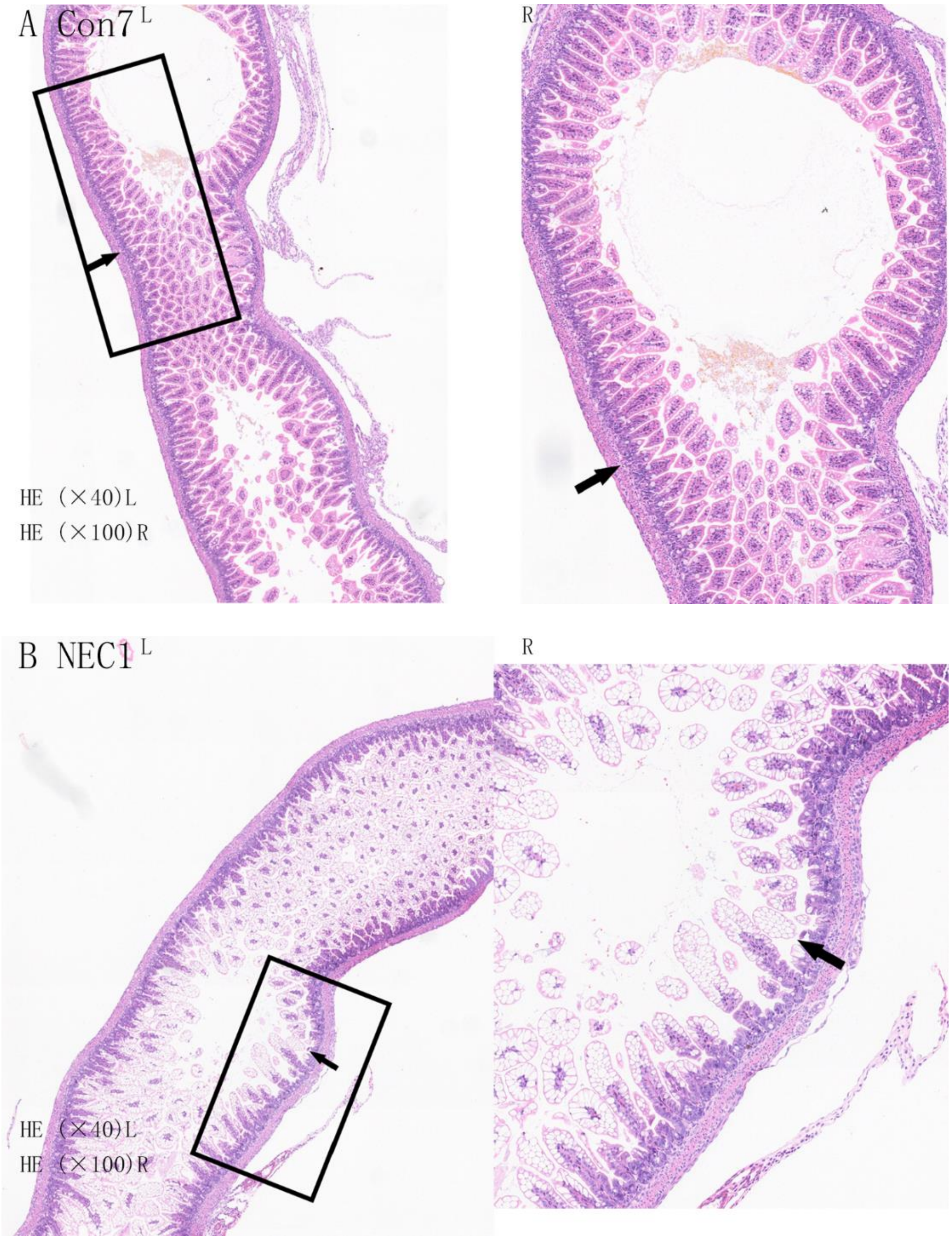
A Con7 L
R
HE (×40)L
HE (×100)R
B NEC1 L
R
HE (×40)L
HE (×100)R

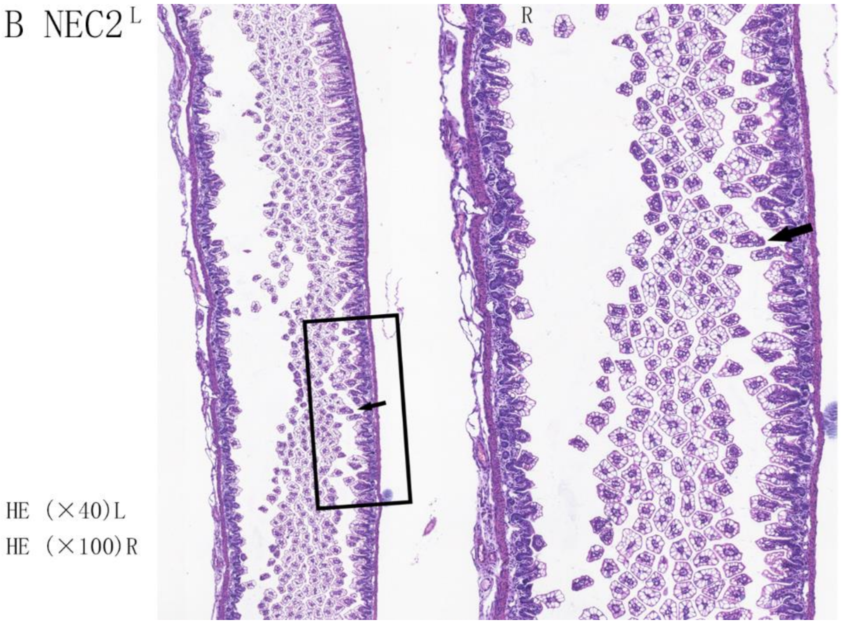
B NEC2 L
R
HE (×40)L
HE (×100)R

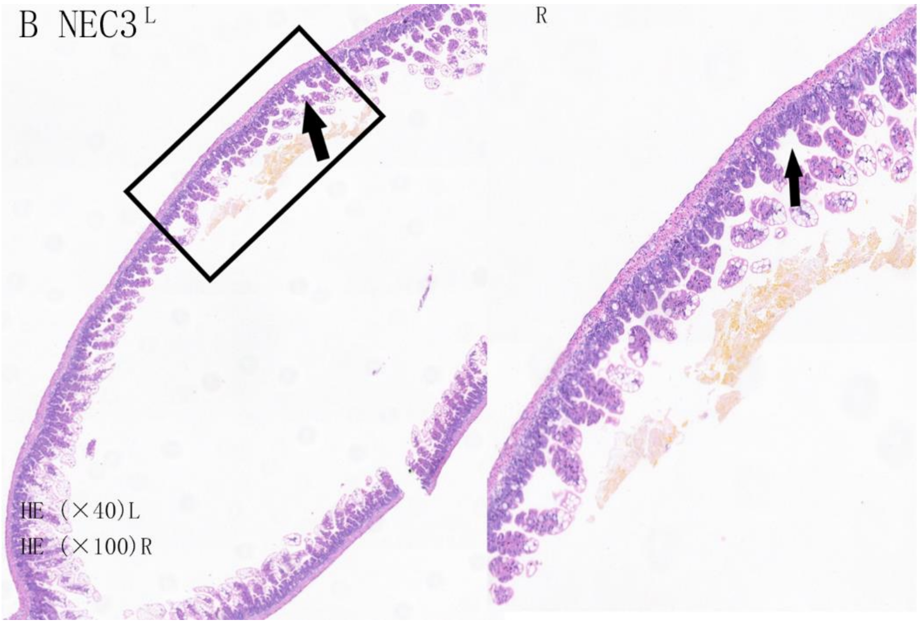
B NEC3 L
R
HE (×40)L
HE (×100)R

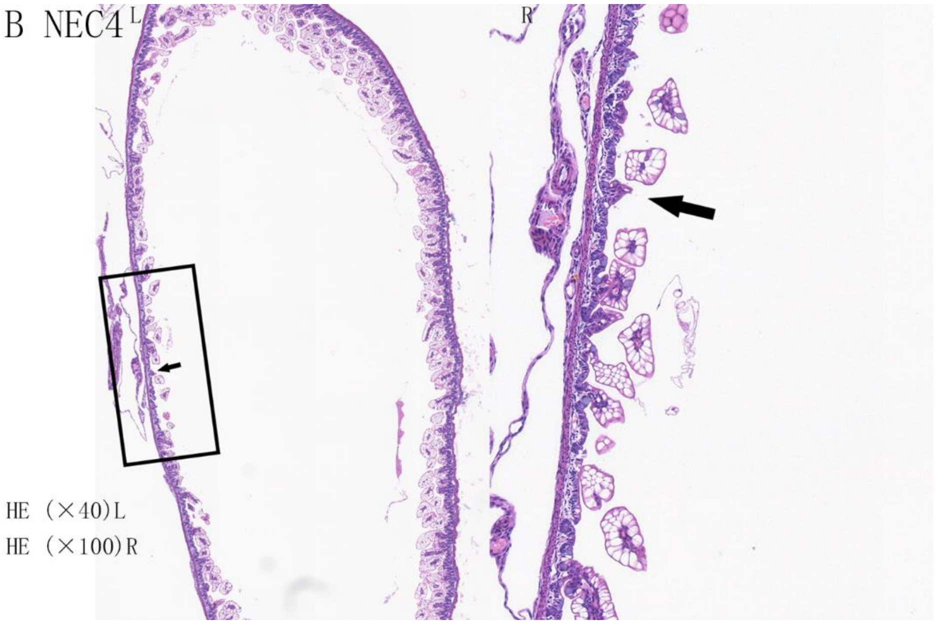
B NEC4 L
R
HE (×40)L
HE (×100)R

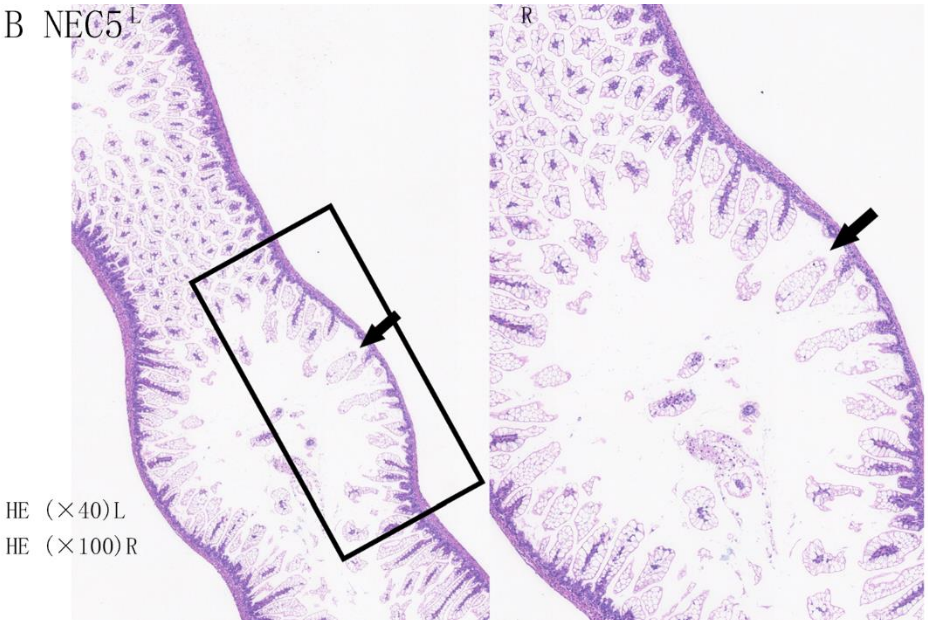
B NEC5 L
R
HE (×40)L
HE (×100)R

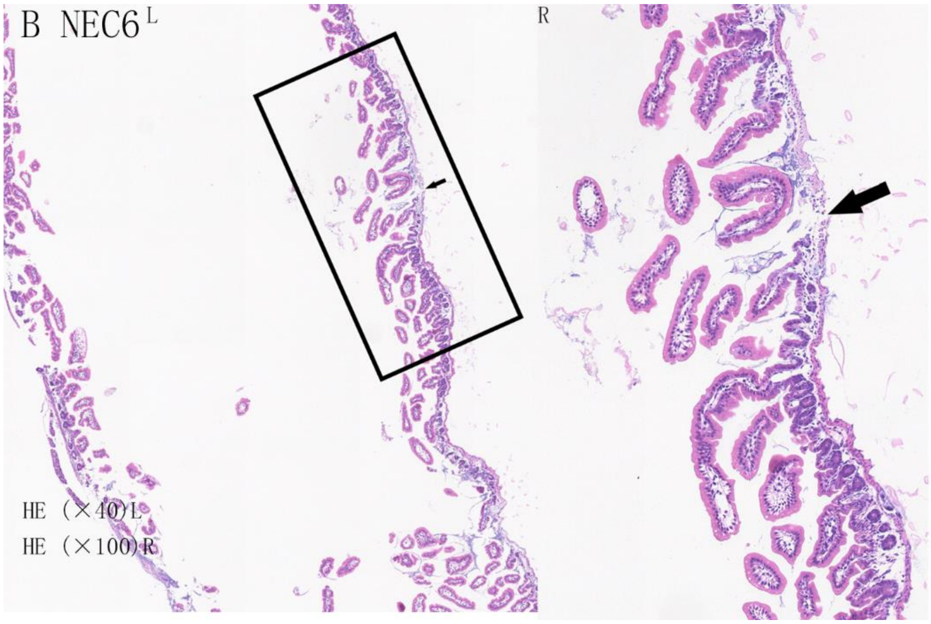
B NEC6 L
R
HE (×40)L
HE (×100)R

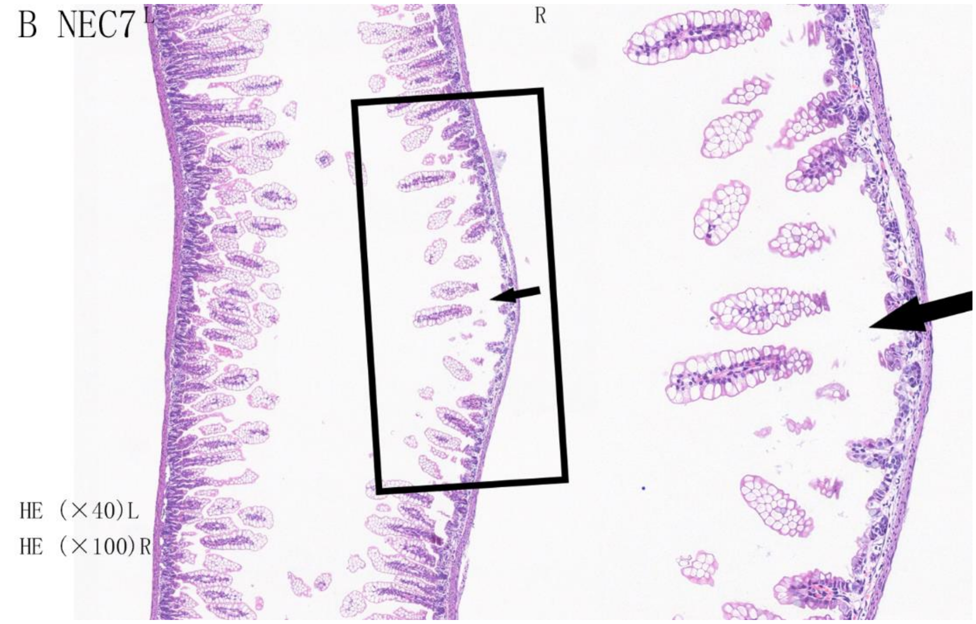
B NEC7 L
R
HE (×40)L
HE (×100)R

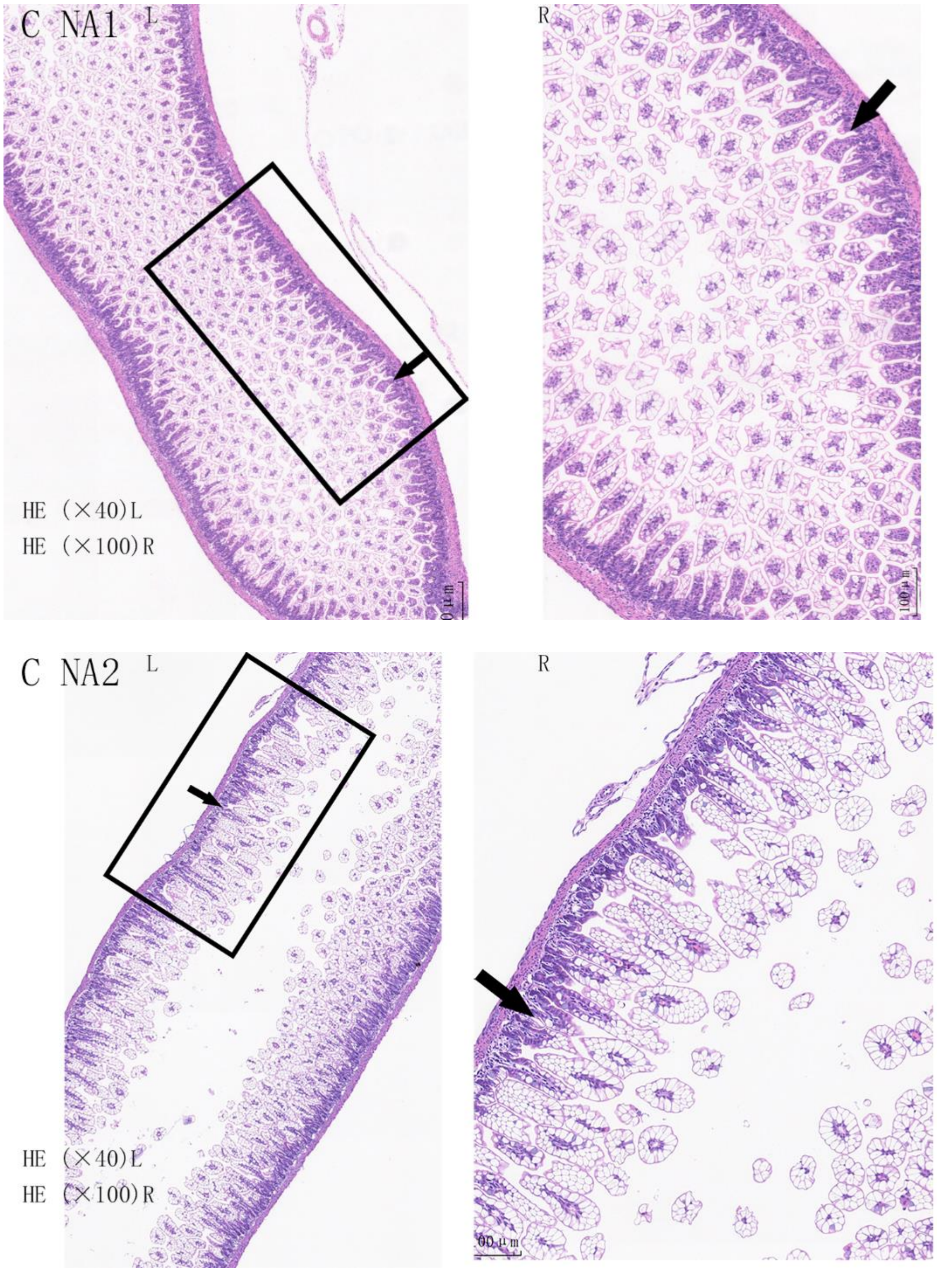
C NA1
L
R
HE (×40)L
HE (×100)R
C NA2
L
R
HE (×40)L
HE (×100)R

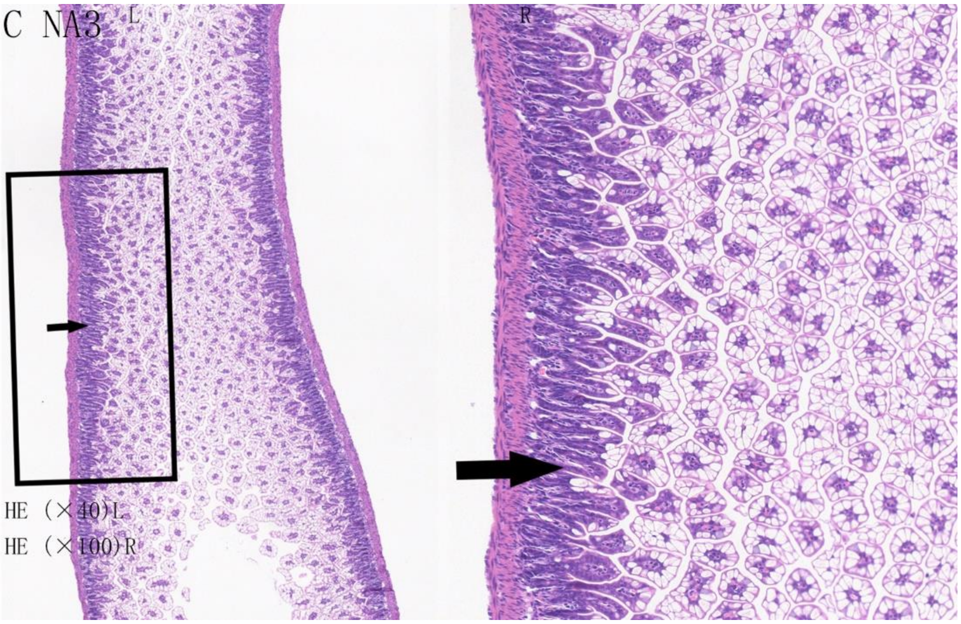
C NA3
L
R
HE (×40)L
HE (×100)R

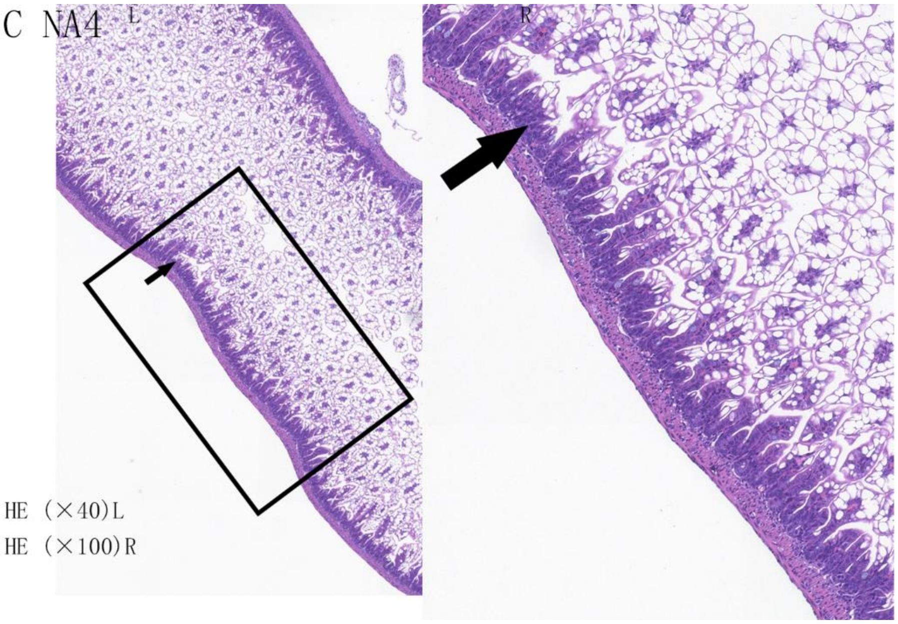
C NA4
L
R
HE (×40)L
HE (×100)R

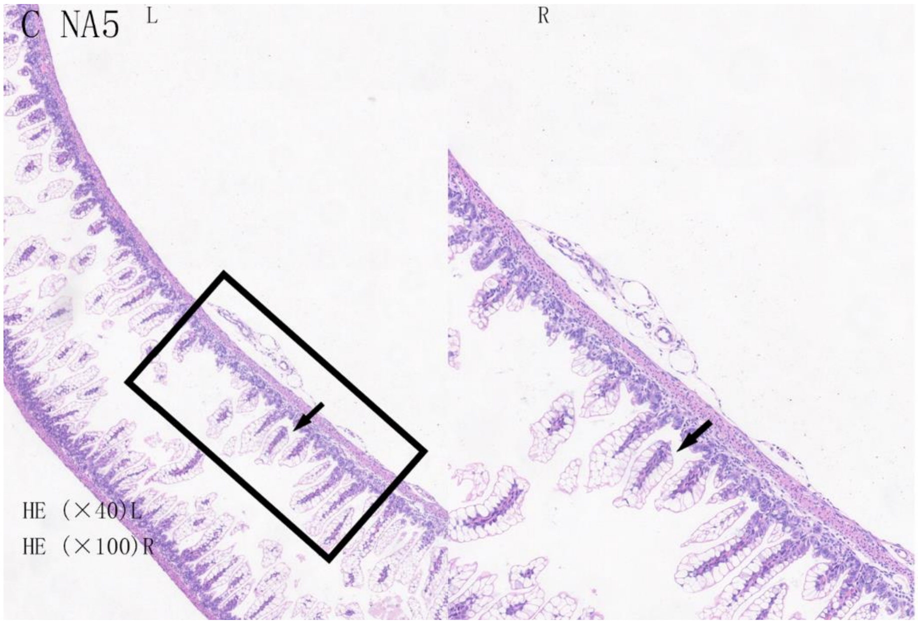
C NA5
L
R
HE (×40)L
HE (×100)R

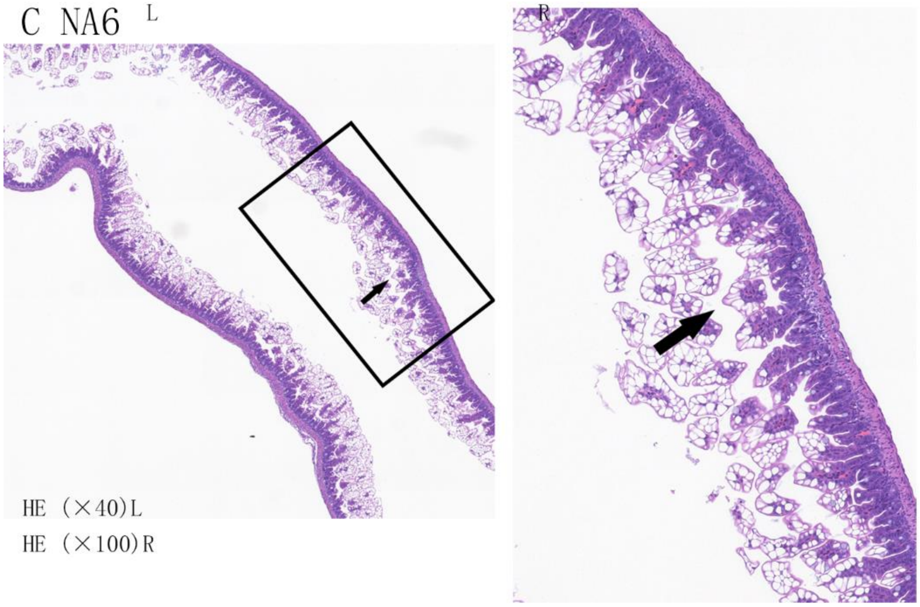
C NA6
L
R
HE (×40)L
HE (×100)R

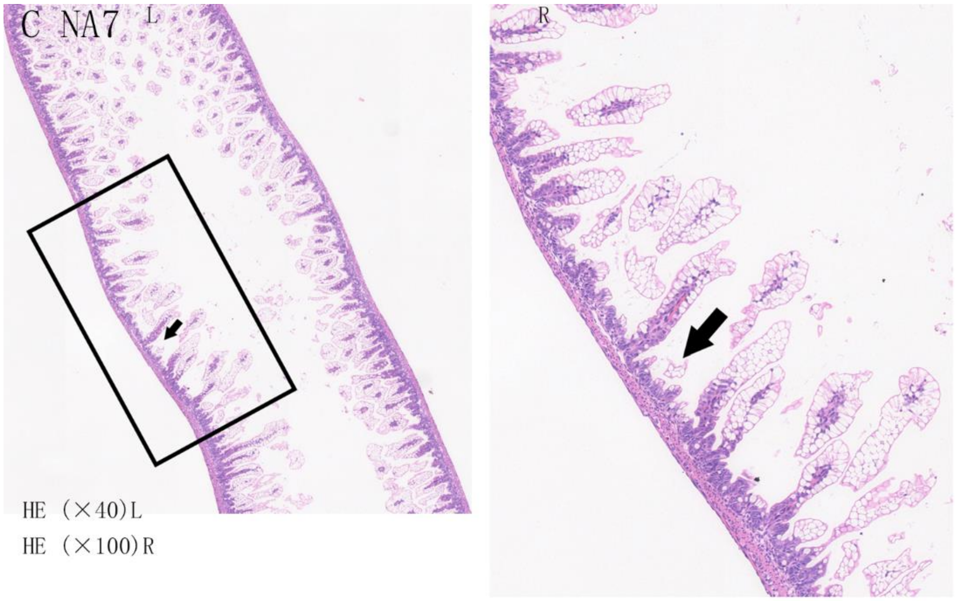

Supplementary Figure 1. (A-C) Light microscopy images of HE staining. Histological damage in the terminal ilea of each sample from the three groups: control group (A, Con1–7), NEC group (B, NEC1–7), NA group (C, NA1–7). Black rectangle indicates a representative area, with a zoomed-in image. Black arrow indicates the damage area. Magnification: ×40 (L-left) and ×100 (R-right). Scale bars: 250 μm (L) and 100 μm (R).
