# Supplementary material for: Exogenous Autoinducer-2 Rescues Intestinal Dysbiosis and Intestinal Inflammation in a Neonatal Mouse Necrotizing Enterocolitis Model
Source: Front Cell Infect Microbiol. 2021 Aug 5;11:694395. doi: 10.3389/fcimb.2021.694395 (PMC8375469; doi:10.3389/fcimb.2021.694395)
Supplement: Supplementary file 2 [file Image_2.pdf]

Supplementary Material

1.1 Supplementary Figures

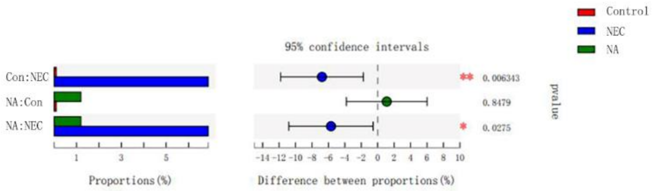

Supplementary Figure 2. Average relative abundances of *Clostridium_sensu_stricto_1* at the species level among the three groups. Numbers of samples: Con (n=12), NEC (n=10), and NA (n=11). Statistics: Kruskal-Wallis test with Scheffe's post-hoc test.
