# Supplementary material for: Exogenous Autoinducer-2 Rescues Intestinal Dysbiosis and Intestinal Inflammation in a Neonatal Mouse Necrotizing Enterocolitis Model
Source: Front Cell Infect Microbiol. 2021 Aug 5;11:694395. doi: 10.3389/fcimb.2021.694395 (PMC8375469; doi:10.3389/fcimb.2021.694395)
Supplement: Supplementary file 3 [file Image_3.pdf]

Supplementary Material

1.1 Supplementary Figures

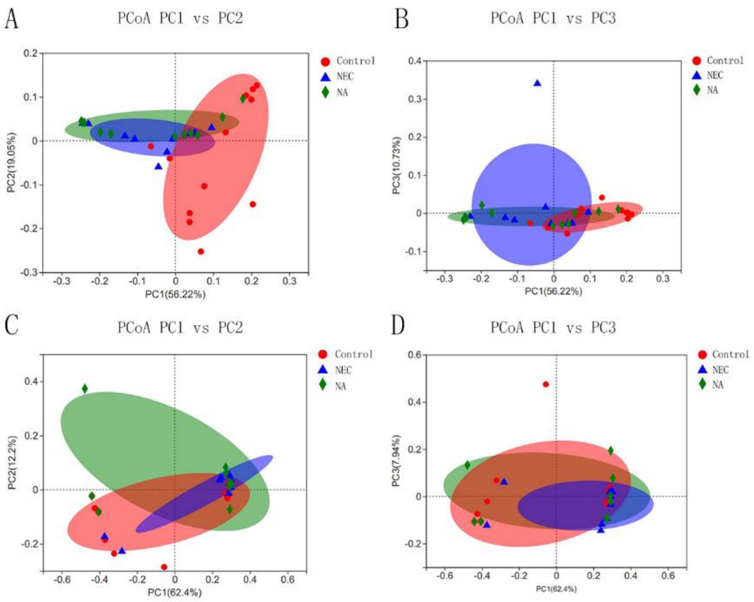

Supplementary Figure 3. PCoA based on weighted and unweighted UniFrac distances. (A and B) PCoA results based on the weighted UniFrac distance. (C and D) PCoA results based on the unweighted UniFrac distance. PC- percent variance explained.
