# Supplementary material for: Exogenous Autoinducer-2 Rescues Intestinal Dysbiosis and Intestinal Inflammation in a Neonatal Mouse Necrotizing Enterocolitis Model
Source: Front Cell Infect Microbiol. 2021 Aug 5;11:694395. doi: 10.3389/fcimb.2021.694395 (PMC8375469; doi:10.3389/fcimb.2021.694395)
Supplement: Supplementary file 4 [file Image_4.pdf]

Supplementary Material

1.1 Supplementary Figures

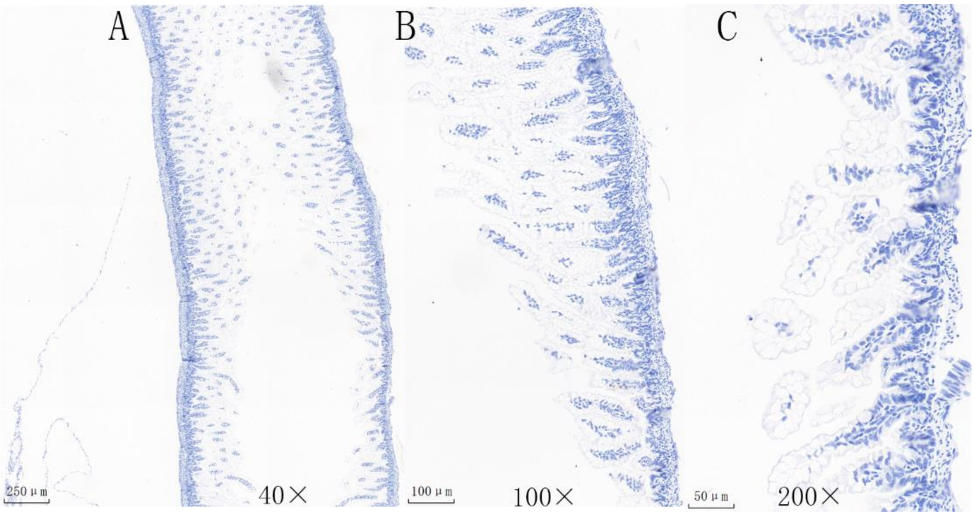

Supplementary Figure 4. Images of immunohistochemical staining (secondary antibody and DAB control alone). (A) Magnification: ×40. Scale bar = 250 μm. (B) Magnification: ×100. Scale bar = 100 μm. (C) Magnification: ×200. Scale bar = 50 μm.
