# Supplementary material for: Exogenous Autoinducer-2 Rescues Intestinal Dysbiosis and Intestinal Inflammation in a Neonatal Mouse Necrotizing Enterocolitis Model
Source: Front Cell Infect Microbiol. 2021 Aug 5;11:694395. doi: 10.3389/fcimb.2021.694395 (PMC8375469; doi:10.3389/fcimb.2021.694395)
Supplement: Supplementary file 5 [file Table_1.docx]

Supplementary Material

# Supplementary Tables 1

## Comparison of bacterial microbiota at the phylum and genus levels before and after AI-2 treatment. Mean ± SD (%)

| Bacterial Microbiota | Control | NEC | NA | *P* |
| --- | --- | --- | --- | --- |
| **Phylum level** |  |  |  |  |
| *Firmicutes* | 62.14±23.16 | 37.79±22.43 | 40.90±29.97 | 0.1274 |
| *Proteobacteria* | 25.26±17.89 | 54.34±25.00 | 58.35±29.77 | 0.0089 |
| *Bacteroidota* | 8.21±9.94 | 0.069±0.08 | 0.1122±0.17 | 0.0001 |
| *Campilobacterota* | 0.60±1.30 | 6.837±19.28 | 0.1684±0.44 | 0.0367 |
| **Genus level** |  |  |  |  |
| *Lactobacillus* | 52.78±23.77 | 7.888±11.03 | 14.36±15.77 | 0.0001 |
| *Escherichia-Shigella* | 14.37±16.29 | 31.26±23.55 | 11.73±12.98 | 0.1251 |
| *Klebsiella* | 1.008±1.90 | 15.24±27.28 | 33.38±34.76 | 0.0023 |
| *Clostridium_sensu_stricto_1* | 0.1108±0.34 | 24.09±24.14 | 21.49±26.71 | 0.0003 |
| *Rodentibacter* | 6.522±12.02 | 6.198±14.24 | 10.02±23.91 | 0.0430 |
| *Helicobacter* | 0.595±1.30 | 6.837±19.28 | 0.1684±0.44 | 0.0434 |
| *Veillonella* | 1.754±3.054 | 3.263±9.23 | 2.239±3.98 | 0.3445 |
| *Streptococcus* | 2.871±3.39 | 0.2113±0.26 | 0.605±0.55 | 0.0186 |
| *norank_f__Muribaculaceae* | 3.175±5.80 | 0.0115±0.01 | 0.03361±0.10 | 0.0084 |
| *Bacteroides* | 2.781±4.96 | 0.0251±0.03 | 0.01973±0.04 | 0.0001 |
| *Muribacter* | 0.4053±0.89 | 0.792±1.21 | 1.164±1.42 | 0.8763 |
| *Clostridioides* | 0.1213±0.20 | 1.462±1.95 | 0.2146±0.53 | 0.5796 |
| *Enterococcus* | 0.136±0.14 | 0.3158±0.30 | 1.323±1.19 | 0.0002 |
| *Morganella* | 0.7505±0.65 | 0.4599±0.94 | 0.5261±1.10 | 0.0802 |
| *Fusobacterium* | 1.542±4.91 | 0.0227±0.04 | 0.0046±0.01 | 0.1584 |

Control – normal control; NEC – necrotizing enterocolitis; NA – necrotizing enterocolitis+AI-2.
