# Supplementary material for: Exogenous Autoinducer-2 Rescues Intestinal Dysbiosis and Intestinal Inflammation in a Neonatal Mouse Necrotizing Enterocolitis Model
Source: Front Cell Infect Microbiol. 2021 Aug 5;11:694395. doi: 10.3389/fcimb.2021.694395 (PMC8375469; doi:10.3389/fcimb.2021.694395)
Supplement: Supplementary file 6 [file Table_2.docx]

Supplementary Material

# Supplementary Table 2

|  | Control | NEC | NA | *P*1 | *P*2 |
| --- | --- | --- | --- | --- | --- |
| Chao  Phylum level | 149.7  (129.3, 196.6) | 113.5  (89.55, 182.1) | 95.25  (83.33, 110.1) | 0.3681 | 0.5737 |
| Chao  Genus level | 66.50  (53.58, 92.14) | 43.85  (34.13, 70.00) | 64.00  (30.00, 76.50) | 0.1106 | 0.7763 |
| Shannon  Phylum level | 2.242  (1.893, 2.782) | 1.605  (1.149, 1.676) | 1.240  (0.9974, 1.873) | 0.0075** | >0.9999 |
| Shannon  Genus level | 1.309  (1.006, 1.864) | 1.075  (0.9694, 1.323) | 1.079  (0.9352, 1.565) | 0.5831 | >0.9999 |
| *P*1: Control *vs.* NEC; *P*2: NEC *vs.* NA | | | | | |

The Shannon and Chao indexes in each group at the phylum and genus levels, M (P25–P75).
